# Genome-wide identification of bZIP gene family and expression analysis of *BhbZIP58* under heat stress in wax gourd

**DOI:** 10.1186/s12870-023-04580-6

**Published:** 2023-11-29

**Authors:** Wei Liu, Min Wang, Min Zhong, Chen Luo, Shaoqi Shi, Yulei Qian, Yunyan Kang, Biao Jiang

**Affiliations:** 1 Guangdong Key Laboratory for New Technology Research of Vegetables, Vegetable Research Institute, Guangdong Academy of Agricultural Sciences, Guangzhou, 510640 Guangdong China; 2 College of Horticulture, South China Agricultural University, Guangzhou, 510642 Guangdong China; 3 Guangdong Laboratory for Lingnan Modern Agriculture, Guangzhou, 510640 Guangdong China

**Keywords:** Wax gourd, Heat stress, Genome-wide identification, Evolutionary pattern, *BhbZIP58*

## Abstract

**Background:**

The basic leucine zipper (bZIP) transcription factor family is one of the most abundant and evolutionarily conserved gene families in plants. It assumes crucial functions in the life cycle of plants, including pathogen defense, secondary metabolism, stress response, seed maturation, and flower development. Although the genome of wax gourd has been published, little is known about the functions, evolutionary background, and gene expression patterns of the *bZIP* gene family, which limits its utilization.

**Results:**

A total of 61 *bZIP* genes (*BhbZIPs*) were identified from wax gourd (*Benincasa hispida*) genome and divided into 12 subgroups. Whole-genome duplication (WGD) and dispersed duplication (DSD) were the main driving forces of *bZIP* gene family expansion in wax gourd, and this family may have undergone intense purifying selection pressure during the evolutionary process. We selected *BhbZIP58,* only one in the member of subgroup B, to study its expression patterns under different stresses, including heat, salt, drought, cold stress, and ABA treatment. Surprisingly, *BhbZIP58* had a dramatic response under heat stress. *BhbZIP58* showed the highest expression level in the root compared with leaves, stem, stamen, pistil, and ovary. In addition, BhbZIP58 protein was located in the nucleus and had transcriptional activation activity. Overexpression of *BhbZIP58* in Arabidopsis enhanced their heat tolerance.

**Conclusions:**

In this study, bZIP gene family is systematically bioinformatically in wax gourd for the first time. Particularly, *BhbZIP58* may have an important role in heat stress. It will facilitate further research on the *bZIP* gene family regarding their evolutionary history and biological functions.

**Supplementary Information:**

The online version contains supplementary material available at 10.1186/s12870-023-04580-6.

## Background

Transcription factors (TFs) play essential regulatory roles in many crucial biological processes in plants, which are classified into different families according to their conserved DNA-binding domains [1–3]. The programmed and regulated interactions between TFs and downstream genes influence metabolic processes and phenotypic traits [2, 4–6]. Currently, about 100 TF families have been identified in Arabidopsis, including MYB, the basic leucine zipper (bZIP), the basic/helix-loop-helix (bHLH), and WRKY [4, 7, 8]. Among them, bZIP transcription factor family is one of the largest and most diverse families in plant [8]. In addition, the bZIP TFs were named after a highly conserved bZIP domain, which contained two structural characteristics on a contiguous helix [8, 9]. Firstly, a basic region of roughly 18 amino acid residues was followed by an invariant N-X7-R/K-X9 motif for nuclear localization and sequence-specific DNA binding. Secondly, the leucine zipper domain was composed of multiple amphiphilic-helices, with one leucine or other hydrophobic amino acid appearing per six amino acids. These amino acids, such as valine, methionine, L-Isoleucine, and L-Phenylalanine, frequently interact via hydrophobic surfaces to create a-helical homodimers or heterodimers [10]. To bind DNA, two subunits engage via the hydrophobic sides of their helices, forming a superimposed coiled-coil shape. In plant, bZIPs preferentially bind to palindromic or pseudo-palindromic ACGT core Cis-regulatory Elements such as G-box (CACGTG), C-box (GACGTC), A-box (TACGTA), and ABRE (CCACGTGG) [11].

Nowadays, bZIP transcription factor family has been identified in model plants such as Arabidopsis, rice, and maize, and many horticulture plants [9, 12–18]. In Arabidopsis, there are 78 bZIP members, which can be divided into 13 groups based on their functional structure (designated A-M) [8, 9]. bZIPs have been linked to responses to a number of abiotic/biotic stimuli, including high salinity, drought, heat, cold, pathogen infection, as well as hormone signaling, including abscisic acid (ABA), ethylene, and light signaling [8, 13]. Specifically, subgroup B of the *bZIP* TFs, namely *bZIP17* and *bZIP28*, exhibit a pivotal function in abiotic stress responses and exhibit a close association with the ABA pathway [19, 20]. Overexpressing *bZIP17* or *bZIP28* in Arabidopsis leads to enhanced tolerance to diverse environmental stresses, whereas their knock-out plants exhibit highly sensitive phenotypes [21–24]. Besides, *AtbZIP17* could collaborate with *AtbZIP28* to regulate development-related genes, particularly those associated with stress maintenance and root elongation [23, 25, 26]. The survival rate and germination rate of *TabZIP28*-overexpressed Arabidopsis were higher than those of wild type after heat stress [27]. In rice, overexpression of *OsbZIP60* (a homologous gene of *AtbZIP17*) could enhance heat and drought resistance [28]. A recent study shown that tobacco plants' tolerance to salt, osmotic, drought, and heat stress was increased by overexpressing the *EcbZIP17* gene from finger millet [29]. Whilst subgroup B of *bZIP* assumes pivotal roles in the intricate tapestry of plant development and stress responses, the molecular function of it under heat stress in wax gourd is unknown.

Wax gourd (*Benincasa hispida* (Thunb.) Cogn, 2n = 2*x* = 24) is an important vegetable crop of Cucurbitaceae family, which has been grown as an annual herb in China for about 1,500 years and has an annual planting area of more than 200,000 hm^2^ [30, 31]. The fruits of wax gourd contain abundant nutrients and metabolites, and can be used as both food and medicine [32, 33]. As a fruit vegetable, wax gourd is sensitive to environmental influences, including sodium chloride, heavy metals, low and high temperatures, and drought stress. Undoubtedly, heat stress is one of the major constraints to crop production and food security worldwide, with a yield loss of 6–7% expected for every 1 °C increase in seasonal mean weather coupled with intense heat stress [34]. High temperature resulted in significant quality and production losses during wax gourd growth, particularly in open field farming [35]. Hence, the heat resistance plays a pivotal role in the breeding of wax gourd. a draft genome sequence of wax gourd was published, serving as a fundamental resource to expedite genetic advancements in this crop [36]. However, up to now, no comprehensive analysis of the *bZIP* gene family in wax gourd has been reported. Consequently, it holds immense importance to systematically investigate the *bZIP* family in wax gourd under heat stress conditions.

In the present study, we identified the *bZIP* gene family based on the wax gourd genome data, and analyzed the phylogeny to determine the relationships between these genes. The gene structure, promoter sequences, and physicochemical properties of the proteins were also analyzed. Subsequently, the subgroup B gene, *BhbZIP58*, was selected for further expression and functional study. Our results provide systematic information on the *bZIP* gene family in wax gourd, which will facilitate the study of function and regulatory mechanisms of *bZIP* family members.

## Results

### Identification of bZIP TFs in wax gourd

**Fig. 1:**
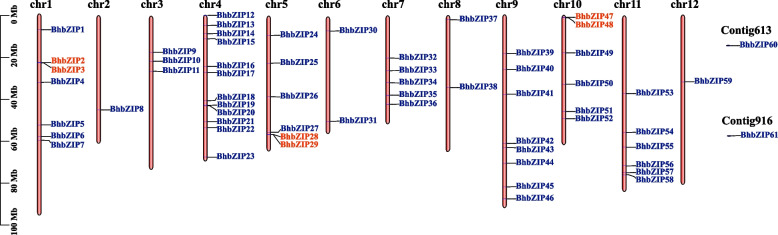
Chromosome distribution of the wax gourd *bZIP* genes. Chr1-12 represent chromosome numbers 1–12 and blue letters represent *bZIP* genes. The gene names in red represented gene clusters

A total of 61 candidate *BhbZIP* genes were identified from wax gourd genome, the nomenclature and related basic information of which were listed in Table S1. Since the bZIP domain of *Bhi09M001946* was severely deleted, so it was not classified in bZIP family. To ensure the accuracy of the results, we also corrected and confirmed each bZIP using the IGV-sRNA tool. The core conserved domain of BhbZIP proteins was discovered to be 50 amino acids long on average, with the bZIP domain comprised of a basic region and a leucine zipper (Fig. 1). According to the position of genes on the chromosome, the *bZIP* genes of wax gourd were designated from *BhbZIP1* to *BhbZIP59*. Besides, two genes, *BhiUN613M6* and *BhiUN916M5*, located on loose scaffolds were named as *BhbZIP60* and *BhbZIP61*, respectively. Chromosome mapping showed that 59 members of *bZIP* family were unevenly distributed on 12 chromosomes (Fig. 1). The chromosome with the most *BhbZIP* genes (12 genes) was chromosome 4, while chromosomes 2 and 12 had only one member. According to a report, two or more genes from the same family that were present in a 200 kb region of a chromosome are considered to be a gene cluster [37]. Three small gene clusters were found based on the BhbZIP location information: *BhbZIP2-BhbZIP3*, *BhbZIP28-BhbZIP29*, and *BhbZIP47-BhbZIP48*. Among the members, *BhbZIP13* had the minimum number of amino acids (136 aa), and *BhbZIP58* had the maximum number of amino acids (763 aa). As shown in Table S1, molecular weight of BhbZIP proteins ranged from 15.71 to 82.68 KDa and the isoelectric point (PI) ranged from 4.45 to 10.56. The average hydrophilicity (GRAVY) of all members of bZIP family was negative, indicating that they were hydrophilic proteins, which was consistent with the potential functions of TFs. Secondary structure prediction results revealed that most BhbZIP proteins were primarily consisted of α-helix and random coil, with the fraction of extended structure and β-turn being the least (Table S2). It was consistent with the ability of the bZIP domain to construct a continuous a-helical structure [9]. The results of subcellular localization prediction showed that all transcription factors located in the nucleus (Table S2).

### Phylogenetic and conserved domain analysis of *BhbZIP* genes

**Fig. 2:**
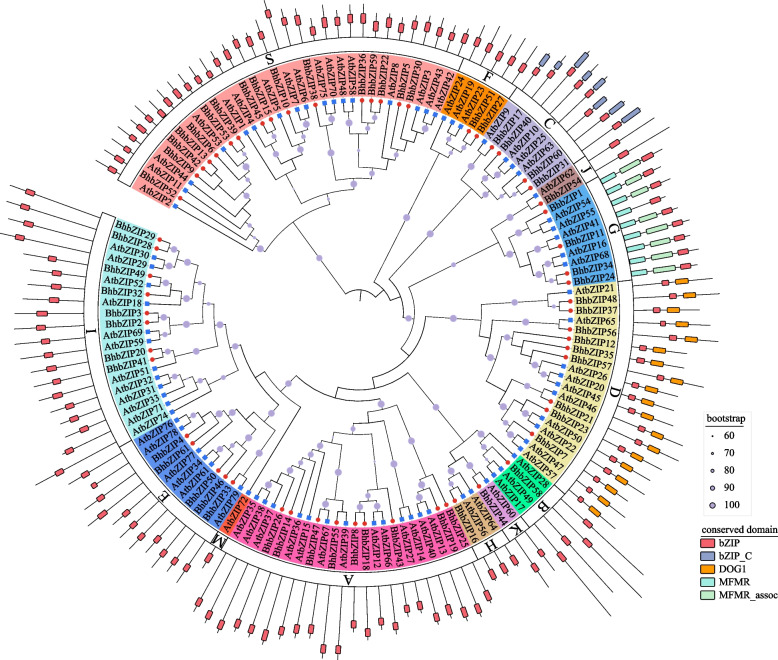
Phylogenetic tree of wax gourd and Arabidopsis bZIP family. Red circles represent wax gourd, blue squares represent Arabidopsis. The dendrogram was drawn by IQ-TREE with the Maximum Likelihood method. Different groups were marked with different colors. The various sizes of circles on the branch represent bootstrap values. The conserved protein domains were represented by the outermost color blocks

To categorize and investigate the evolutionary relationship of the *BhbZIP* genes, a phylogenetic tree was created using the bZIP proteins of wax gourd and Arabidopsis. Phylogenetic analysis showed that the *BhbZIP* genes were divided into 12 subgroups (A, B, C, D, E, F, G, H, I, J, K, S) based on the relationship with *AtbZIPs* (Fig. 2). We found that the short sequences of wax gourd and Arabidopsis were concentrated in subgroup S and the long sequences were concentrated in subgroup B. The number of wax gourd and Arabidopsis bZIPs in each subgroup was similar approximately. Among the subgroup, the subgroup M was deleted and only one *bZIP* gene was identified in subgroup B, J, H, and K, while the largest group was the subgroup S with 16 *BhbZIP* genes. Furthermore, we noticed that several subgroups of wax gourd contained the unique domain, with group G, D, and C having MFMR and MFMR_assoc domains, DOG1 domain, and bZIP_C domain, respectively.

### Exon/intron structures and conserved motifs of *BhbZIP* genes

**Fig. 3:**
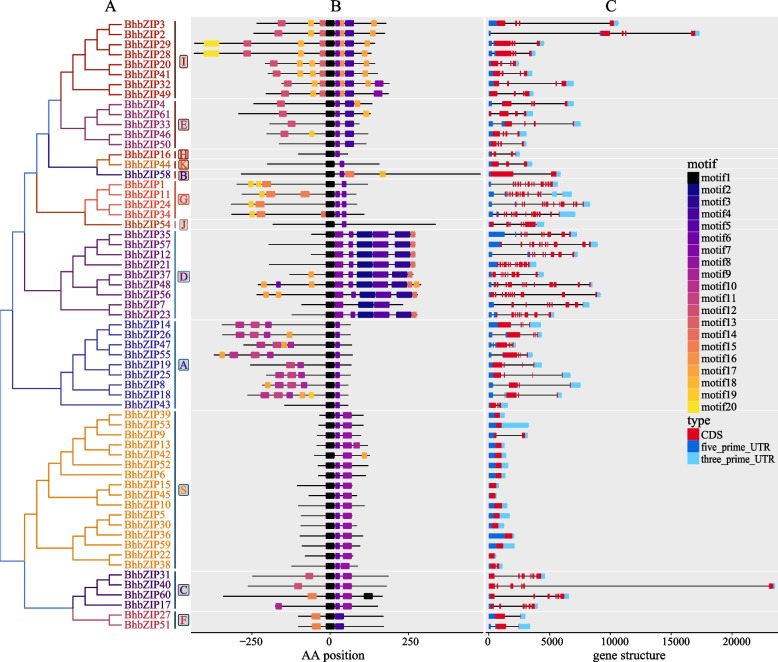
Gene structures and protein motifs of *bZIP* gene family in wax gourd. **A** Phylogenetic tree of wax gourd *bZIP* gene family. Different colors represented different subgroups, and the majuscule was the name of the subgroup. **B** Protein motifs in bZIP members. The colorful boxes delineated different motifs, and the black box (motif1) represented bZIP domain. **C** Gene structures of wax gourd *bZIP* gene family. Exons, 5’ UTR, and 3’ UTR were respectively displayed using red, dark blue, and light blue rectangles. Black lines denoted introns. The clustering was performed according to the results of phylogenetic analysis

To understand the conserved structure and evolution of wax gourd bZIP family, we discovered 20 conserved motifs using MEME analysis combined with a phylogenetic tree (Fig. S2). Motif1 was a basic region of the bZIP structural domain, while the leucine zip was not very conserved and was distributed in motif4, motif6 and motif7. The number of leucine repeats in the ZIP domain varied considerably, from 3 times (Group D) to 8 times (Group C and group S). Motif2 was the DOG1 domain in the Pfam databases. As shown in Fig. 3B, all of the bZIP members had motif1. In contrast, motif2, motif5, and motif3 were present only in Group D. Similarly, motif10 and motif11 were only found in Group A. Many motifs existed in a specific subgroup, which might be related to specific biological functions.

To ensure the accuracy of the bZIP gene structure, we corrected and complemented them using transcripts. Most *BhbZIP* genes in the same subgroups had a similar exon–intron structure (Fig. 3C). For instance, the genes of subgroup S contained only one or two exons, while those of subgroups D and G had a high number of exons. Besides, all the members of Group G had two five-prime untranslated regions (UTR).

### Intra- and inter-species collinearity analysis of *BhbZIP* genes

**Fig. 4:**
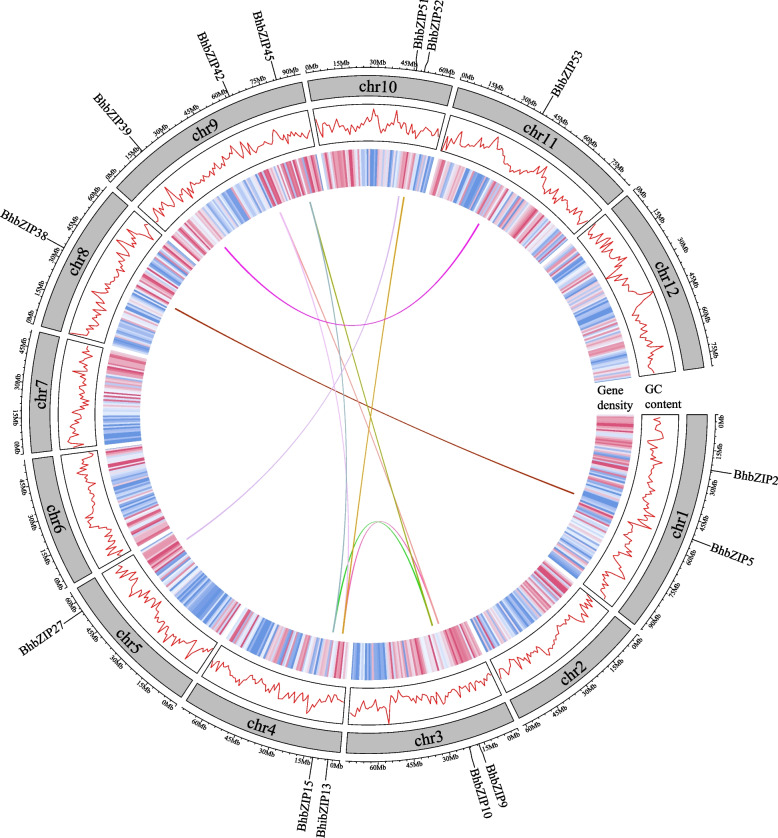
Collinearity analysis of bZIP family in wax gourd. Chromosomes 1–12 were represented by grey rectangles. The different color lines and heatmaps along the rectangles respectively represented GC content and gene density on the chromosomes. The different colour lines represented collinear gene pairs of *BhbZIPs* between the wax gourd chromosomes

The members of *bZIP* family in wax gourd were generated through four replication events, with no tandem duplication existed (Table S4). Based on the analysis of gene replication events, the number of *BhbZIP* genes generated by Dispersed, WGD, Transposed, and Proximal was 30 (51%), 14 (24%), 11 (18%), and 4 (7%), respectively. On the chromosomes of wax gourd, a total of 10 collinear gene pairs of *bZIP* genes were found (Fig. 4), and listed in Table S5. Nine of them were from subgroup S and only one was from subgroup F, and all genes from collinearity were generated by WGD replication events. Especially, there were two collinear pairs consisting of three genes that had collinearity relationships with each other. Two small clusters of *bZIP* genes, *BhbZIP2-BhbZIP3* and *BhbZIP28-BhbZIP29*, formed two groups of proximal replicates with sequence similarity bigger than 70%, and all belonged to Group I. Furthermore, we calculated the Ka/Ks ratios of 12 sets of *BhbZIP* genes to explore the evolutionary selection pressure. The result showed that all the Ka/Ks values of collinear gene pairs of *BhbZIP* were lower than 1, which indicated that these genes were subjected to purified selection (Table S5).

To explore the collinearity relationship between wax gourd and other species, three dicotyledons (Arabidopsis, grape, and cucumber) and one monocotyledon (rice) were chosen (Fig. S3). A total of 49 collinear pairs were found between wax gourd and Arabidopsis. Among them, some *BhbZIP* genes had collinearity relationships with two to three genes of Arabidopsis, suggesting that these genes were crucial in the evolution of *bZIP* family in wax gourd. The number of collinearity gene pairs of wax gourd vs cucumber, and wax gourd vs grape, were 35 and 60, respectively (Table S6). However, only 11 collinearity pairs were found between rice and wax gourd. According to Venn diagram (Fig. S3), four of the BhbZIP pairs were collinear with the four species. Additionally, a total of 11 collinear gene pairs were identified between wax gourd and cucumber/grape/Arabidopsis species, while no found in the rice genome.

### Cis-regulatory elements analysis of *BhbZIP* promoters

**Fig. 5:**
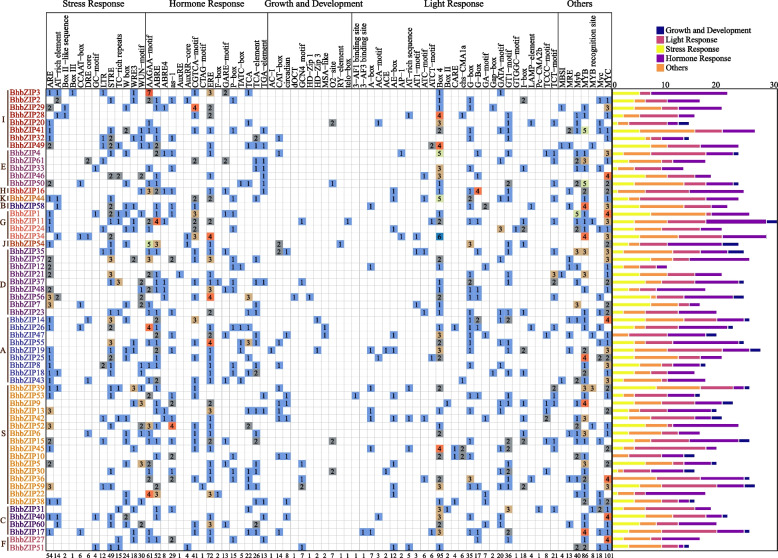
Analysis of Cis-regulatory Elements in *BhbZIP* promoters. The different colors and numbers of grids indicated the numbers of different Cis-regulatory Elements. The numbers at the bottom of the graph represented the total number of Cis-regulatory Elements in *BhbZIP* promoters. The histograms with different colors on the right represented the sum of the Cis-regulatory Elements in each category

To better understand how bZIPs participated in the regulation of biotic and abiotic stresses, the 2000 bp upstream promoter sequences of the *BhbZIP* genes were used to search the Cis-regulatory Elements using PlantCARE online software. Cis-regulatory Elements were classified into 5 categories according to their functions (Fig. 5). The *BhbZIP* promoter region contained lots of hormone response elements, including abscisic acid (AAGAA-motif, ABRE, ABRE4, F-box), methyl jasmonate (CGTCA-motif), gibberellin (GARE-motif, P-box, TATC-box), salicylic acid (TCA, TCA-element, as-1), ethylene (ERE), jasmonic acid (CTAG-motif), auxin (AuxRE, AuxRR-core, TGA-element), which was consistent with the function of bZIP transcription factor in response to abiotic stress. They also contained many stress response elements such as heat stress related elements (STRE, CCAAT-box, AT-rich), cold stress response elements (LTR), anaerobic response elements (ARE), damage and defence response elements (WRE3, WUN-motif or W box). In this study, we also found a large number of functional elements related to light response, such as Box 4, G-box, GT1-motif, I-box, and so on, which was consistent with previous reports of bZIP regulation of photosynthesis [9]. A few *BhbZIP* genes also contained regulatory elements related to growth and development, such as endosperm expression-related elements (GCN4), meristems expression-related elements (CAT-box), seed expression-related elements (RY-element), differentiation-related elements (HD-Zip1), zein metabolism regulatory related elements (O2-site), circadian regulatory elements (circadian). Interestingly, almost all *BhbZIP* genes contained cis-elements of MYB and MYC. Conducting a thorough analysis of the promoter region of the *BhbZIP* gene family contributes significantly to the profound exploration of their intricate biological functionalities.

### protein–protein interaction network of BhbZIP proteins

**Fig. 6:**
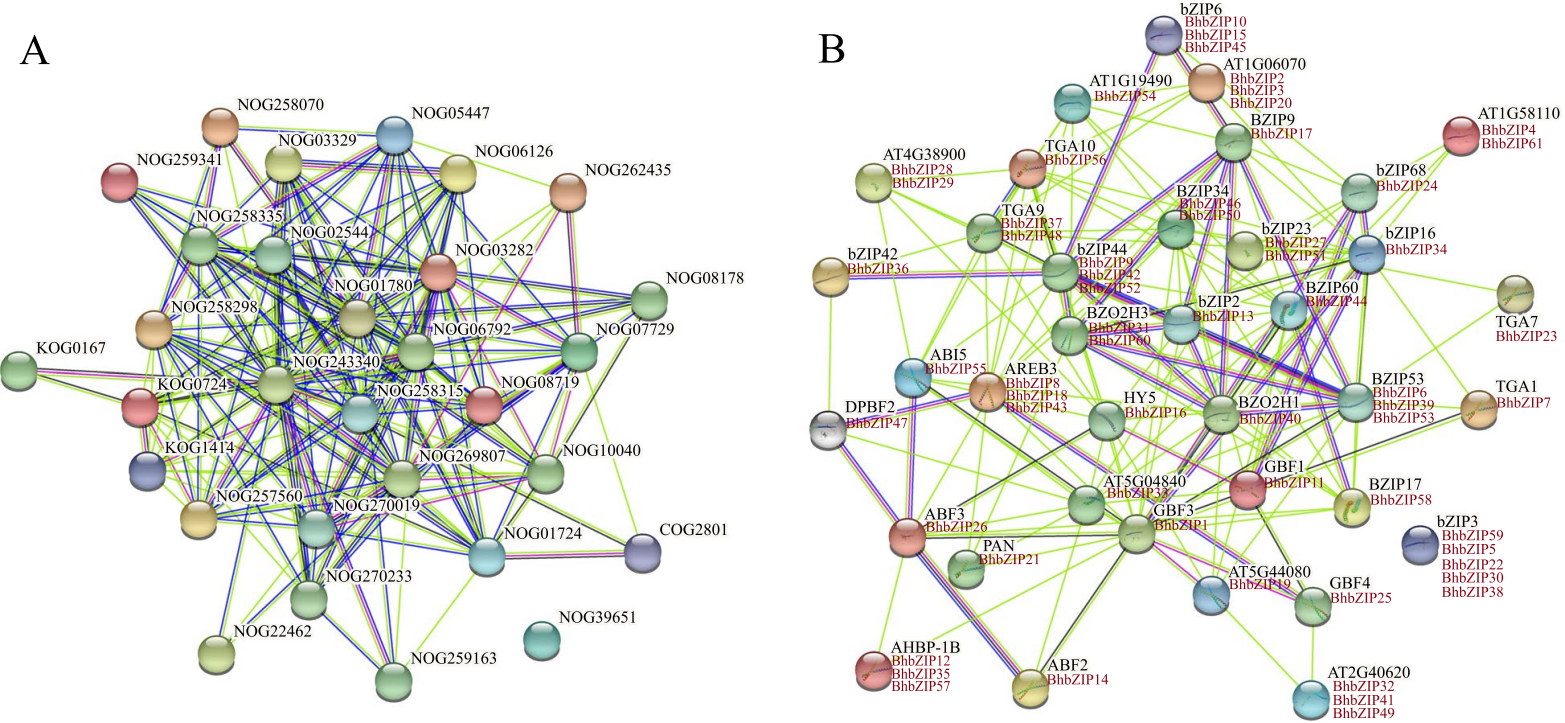
Interaction network of BhbZIP proteins. **A** Search for the orthologs in Arabidopsis matching BhbZIPs. **B** The BhbZIP protein–protein interaction network. This network was predicted by the online software STRING. The BhbZIP proteins were shown in the red font below the Arabidopsis orthologs. Purple lines indicated experimentally validated protein interactions; green lines indicated predicted protein interactions based on gene neighborhood; blue lines indicated predicted protein interactions based on gene co-occurrence; and black lines indicated protein coexpression

Based on studies of AtbZIP and gene homology, we could speculate about the potential functions of most BhbZIP proteins. An interaction network was constructed by using the STRING database based on the search for protein families (Fig. 6A) and multiple sequences (Fig. 6B). The result showed that 12 members were involved in the ABA activation signal pathway (NOG243340), 7 of which belonged to subgroup A, and 5 belonged to subgroup S (Table S3). In the growth and development processes, *BhbZIP55* (subgroup A), *BhbZIP40* (subgroup C), and *BhbZIP21* (subgroup D) involved in seed germination (NOG257560), seed maturation (NOG10040), and flower development regulation (NOG259341), respectively.

Some bZIP genes were associated with other members. *BhbZIP1* was linked with several genes including *BhbZIP14*, *BhbZIP26*, *BhbZIP55*, *BhbZIP6*, *BhbZIP39*, *BhbZIP53*, and *BhbZIP7* (Fig. 8B). *BhbZIP40* was in the core region and interacts with several genes, such as *BhbZIP13*, *BhbZIP17*, *BhbZIP52*, *BhbZIP42*, and *BhbZIP9*. There was an interaction between *AtbZIP17*, a heat stress-related transcription factor, and *AtbZIP60*, and their homologous protein in wax gourd, *BhbZIP58*, was linked to *BhbZIP60* by a purple line, suggesting that they might interact.

### Expression patterns of* BhbZIP58* in different tissues and abiotic stress

**Fig. 7:**
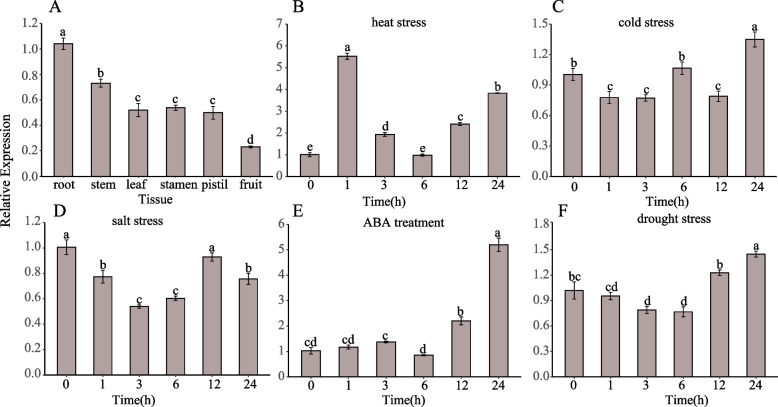
Expression level of *BhbZIP58* in different tissues (**A**), under heat stress (**B**), cold stress (**C**), salt stress (**D**), ABA treatment (**E**), and drought stress (**F**). Data were means ± SEM of three biological replicates, and the transcription level of *BhbZIP58* at 0-24 h and different tissues were statistically evaluated by Duncan's test. Different letters meaned significant differences at 0.05 levels

We randomly selected 12 *BhbZIPs* from the A-S subgroups and conducted expression analysis under heat stress. Remarkably, our findings revealed that *BhbZIP58* exhibited significant induction to heat stress rapidly (Fig. S4). In Arabidopsis, the pivotal role of subgroup B within the bZIP gene family in abiotic stress has been well-documented [20, 21, 25, 26]. However, there is no relevant literature or reports regarding the involvement of subgroup B of the bZIP gene family in abiotic stress in wax gourd. So, we chose the subgroup B member of wax gourd (*BhbZIP58*) to study its expression patterns in various tissues obtained from plants at the reproductive stage under normal growing circumstances by the real-time quantitative PCR (qRT-PCR). As shown in Fig. 7A, *BhbZIP58* was expressed in all organs, with the highest expression in root and the lowest expression in fruit.

To explore the expression of *BhbZIP58* in different stresses, firstly, we wondered if *BhbZIP58* was influenced by the circadian clock. We sampled seedlings under normal growth conditions and performed qPCR assay on *BhbZIP58* with the circadian-regulated gene *LHY* (*Bhi12G002045*) as control. Results showed that there was no significant change in the expression of *BhbZIP58* among the 24 h (Fig. S5), indicating thar *BhbZIP58’s* expression with insusceptible by circadian rhythms. Next, we performed five treatments including high temperature, low temperature, salt stress, drought, and ABA to detect *BhbZIP58* expression. When exposed to high temperature stress, *BhbZIP58* was up-regulated and had two expression peaks, with the highest peak at 1 h and the second peak at 24 h (Fig. 7B). In the process of cold stress, salt stress and drought stress, the expression of *BhbZIP58* had a similar trend, which decreased at first and increased at 3 h or 6 h. For ABA treatment, the expression was almost unchanged at the beginning, but rose sharply at 12 h and reached to the peak at 24 h (Fig. 7E).

### Subcellular localization and transcriptional activity analysis of *BhbZIP58*

First of all, we used the primers ranging from the start codon to the stop codon to clone *BhbZIP58* and found that it could not be cloned. Subsequently, we compared its protein sequence with the homologues of other cucurbits and found that the gene had a redundant sequence, possibly due to an error in the genome annotation file (Fig. S6). Consequently, we designed two primers to clone *BhbZIP58* based on the corrected sequence (Fig. S7).

**Fig. 8:**
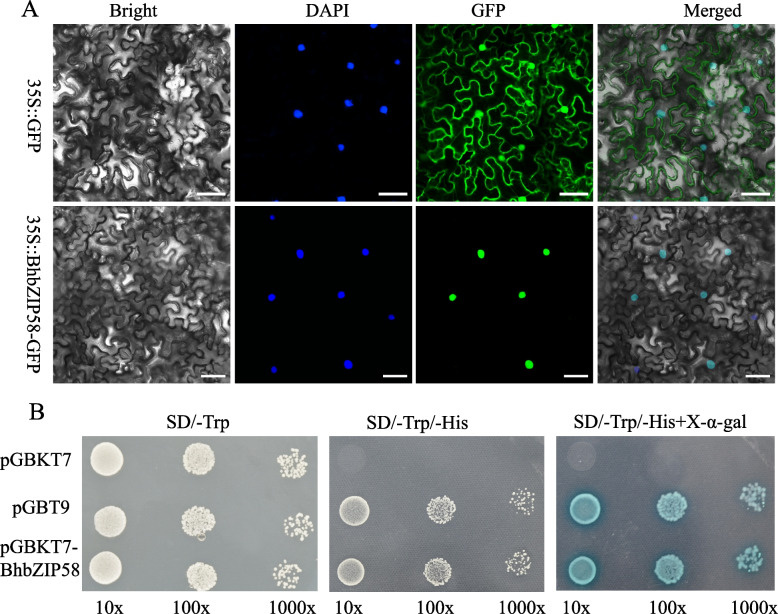
Subcellular localization and transcriptional activation assay of BhbZIP58 protein. **A** Subcellular localization of the 35S::*BhbZIP58*-GFP fusion protein in tobacco leaves. Free GFP served as a control. A DAPI staining assay was conducted to confirm the nuclear localization. White bars = 25 μm. **B** Growth of yeast cells (strain AH109) transformed with pGBT9 vector (a positive control), pGBKT7 vector (a negative control), or pGBKT7-BhbZIP58 vector on SD/-Trp or SD/-Trp/-His with or without X-α-gal. 10 × , 100 × , and 1000 × represent yeasts that were diluted with the original concentration for 10-, 100-, and 1000- fold, respectively

The fluorescent protein (GFP) tag was fused to the C terminus of BhbZIP58 under the control of the super promoter to determine the subcellular localization. *Agrobacterium tumefaciens*-mediated transient expression was used to transfer the 35 s:*BhbZIP58*-GFP recombinant construct and the control into tobacco leaves. The result showed that BhbZIP58-GFP fusion protein was found only in the nucleus (Fig. 8A). To investigate whether *BhbZIP58* acted as a transcriptional activator, transcriptional activation assays were performed. As shown in Fig. 8B, BhbZIP58 protein exhibited self-activation, as the yeast AH109 cells transformed with the pGBKT7-BhbZIP58 and pGBT9 (PC) grew well on the SD/-Trp/-His medium and turned blue on the selective medium containing X-α-Gal.

### Functional analysis of the *BhbZIP58* gene under heat stress

**Fig. 9:**
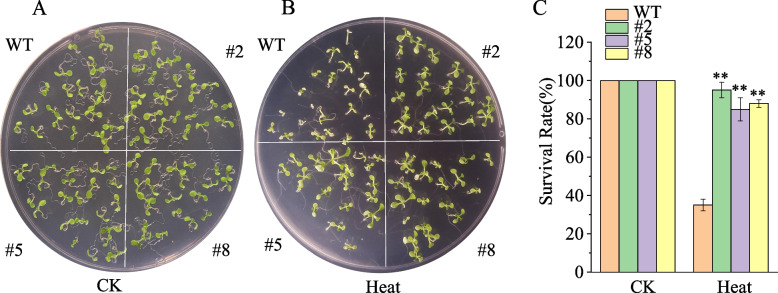
Functional analysis of *BhbZIP58* under heat stress. **A**-**B** Overexpression of the *BhbZIP58* gene in Arabidopsis under different heat stress. **C** Survival statistics of overexpression lines and WT under heat stress. Asterisks indicate significant differences between the transgenic lines and WT according to Student’s t-test (*n* = 3, **P < 0.01)

To better understand the biological role of *BhbZIP58* under heat stress, we transferred it into Arabidopsis for heterologous overexpression. We generated a 35S:*BhbZIP58* construct and transformed it into Arabidopsis. The expression of *BhbZIP58* in the transgenic lines was significantly higher than that in the wild type (Fig. S8). All transgenic lines displayed similar phenotypes under heat stress. The overexpression strain and the wild type grew identically under normal conditions (Fig. 9A). After heat stress treatment, most wild-type plants were visibly stressed, with thin plants, yellowing leaves and even death, but overexpressed plants remained healthy and only 10% died. (Fig. 9B, C). These results indicated that *BhbZIP58* could improve the heat tolerance.

## Discussion

The *bZIP* genes play crucial roles in regulating plant growth, development, and response to biotic and abiotic stresses including high temperature, drought, salt, cold, and ABA treatment [2, 8, 9]. So far, *bZIP* gene family has been reported in several plant species, such as Arabidopsis, rice, maize, tomato, and cucumber [8, 12–14, 17]. However, no study on the *bZIP* gene family of wax gourd has been reported. In this study, we systematically studied the bZIP gene family in wax gourd. A total of 61 *bZIP* genes were identified from wax gourd genome, which distributed unevenly on the 12 chromosomes (Fig. 1). The number of *bZIPs* in wax gourd was similar to that of cucumber, watermelon, pomegranate and poplar, which might be caused by ancient polyploid events [14, 16, 38, 39]. For the accuracy of the follow-up results, we corrected and confirmed each *BhbZIP* gene using the transcriptome data of our research group. The basic biochemical properties (MW, PI, GRAVY, secondary structure, subcellular localization) of the BhbZIP protein sequences were predicted (Tables S1, and S2). Then we analyzed the BhbZIP proteins by multiple sequence alignment and found that the BhbZIP proteins had two basic domains: a highly conserved DNA-binding basic region and a diverse leucine zipper, which was consistent with that of other plants, such as Arabidopsis, rice, maize [12, 14, 15, 40].

The classification and functional research of bZIP family in Arabidopsis had been extensively studied, which were divided int 13 subgroups based on their function and structure [9]. To further understand the evolutionary relationships between Arabidopsis, a phylogenetic tree of *BhbZIP* and *AtbZIP* genes was constructed. Then the 61 *BhbZIP* genes were divided into 12 subgroups according to the classification criteria and genetic relationship of Arabidopsis, which was different from cucumber and watermelon [14, 38] (Fig. 2). When compared with Arabidopsis, the subgroup M of wax gourd was deleted and some subgroups had relatively smaller numbers of members, which implied that *BhbZIP* might lose during the evolution process of wax gourd. The short sequences of *AtbZIP* and *BhbZIP* were concentrated in subgroup S and the long sequences were concentrated in subgroup B. These findings showed that the conserved regions of these genes had a similar evolutionary relationship between wax gourd and Arabidopsis. Subsequently, we predicted the motifs of BhbZIP protein sequences on the MEME website and obtained the gene structure of *BhbZIPs* from wax gourd reference genome. The result showed that the same subgroup had similar motif distribution patterns and gene structures, which was similar to that of other plants (Fig. 3) [14, 16, 38]. The foundational bZIP core domain, known as motif1, exhibited its presence across all BhbZIP proteins, while certain distinctive motifs were exclusive to specific subgroups. This intriguing observation suggested the BhbZIP might assume an indispensable role, considering the diverse functions attributed to bZIP.

Previous studies showed that during the evolution process, gene families typically underwent tandem duplication or large-scale segmental duplication events [41]. Since the *bZIP* gene family was relatively large, we analyzed the tandem duplication events and segmental duplication events of *BhbZIP*s. Interestingly, we found no tandem duplication events existed in *bZIP* genes of wax gourd. Meanwhile, the results of DupGen_finder implied that whole-genome duplication (WGD) and dispersed duplication (DSD) were the main driving forces of *bZIP* gene family expansion and evolution in wax gourd. Nine of the collinear gene pairs were discovered from the subgroup S and only one from the subgroup F, implying that the reason for the large number of members in the subgroup S might be due to chromosomal segmental duplication. Moreover, the values of non-synonymous (Ka) and synonymous (Ks) and the ratio of non-synonymous to synonymous substitutions can be used to evaluate whether a protein-coding gene was under selective pressure [42]. The calculated Ka/Ks ratio of all *BhbZIP* gene pairs was less than 1, which indicated that these genes might have experienced strong purifying selection pressure with limited function divergence during evolution. Canon's study showed that collinearity analysis implied that sequences from two species from the same ancestral sequence had similar functions [43]. To investigate the evolution of bZIP family of wax gourd, we examined the collinearity analysis of *bZIP* genes from other species, such as cucumber, Arabidopsis, grape, and rice (Fig. S3). Our results showed that more *BhbZIP* collinear gene pairs existed in Arabidopsis and grape than cucumber, which might be due to the loss of *bZIP* genes during the evolution of Cucurbitaceae. Some BhbZIP genes exhibited multiple collinearity pairs with other species, suggesting their potential significance in evolution, potentially as ancestral genes. While only a few collinear gene pairs were found in wax gourd vs rice, probably due to the dramatic divergence of bZIP in dicotyledonous and monocotyledonous plants, which was similar to the result of potato [18]. According to Venn diagram (Fig. S3), four of the *BhbZIP* gene pairs were collinear with the four species, suggesting that these genes might have existed before these species diverged and have remained collinear ever since. Of note, a total of 11 collinear gene pairs were identified between wax gourd and cucumber/grape/Arabidopsis species, while no found in the rice genome, this suggested that these gene pairs might have emerged after dicotyledones and monocots differentiation.

Plant bZIP proteins exhibit a relaxed DNA-binding specificity for DNA sequence motifs containing an ACGT core [11]. We predicted the Cis-regulatory Elements of the BhbZIPs promoter and identified many ACGT-containing promoter elements, such as G-box and ABRE. Some cis-elements were associated with hormone regulation, including auxin, gibberellin, abscisic acid, and methyl jasmonate responsive elements, and some were induced by environmental stress, including ARE, STRE, LTR, and WUN-motif, which was consistent with previous reports [9, 42]. MYB and MYC elements that participate in broad range of stresses like drought, salinity and low-temperature existed in almost all the *BhbZIPs* [44]. However, only a small number of regulatory elements related to growth and development existed in *BhbZIPs*. A protein interaction network indicated the potential interactions and co-expression of different *BhbZIPs*, which corresponded to the formation of dimers between bZIP proteins [9]. Interestingly, the heat shock elements (HSE), which often existed in the promoters of numerous genes induced by heat stress, were didn’t found in *BhbZIPs* promoters. There are two members, *bZIP17* and *bZIP28*, in the subgroup B of the Arabidopsis bZIP family, while the corresponding subgroup B of wax gourd was only *BhbZIP58*. Phylogenetic analysis showed that BhbZIP58 had high homology with *AtbZIP17*. Therefore, we hypothesized that the BhbZIP58 gene in wax gourd also has similar function to the Arabidopsis. In this study, we analyzed the response of *BhbZIP58* to 5 kinds of stresses (Fig. 7B-F). The results showed that *BhbZIP58* responded to all the 5 stresses, especially to heat stress. In addition, bZIP17/28 in Arabidopsis were involved in the ABA pathway [25]. Interestingly, *BhbZIP58* expression was up-regulated after ABA treatment, indicating that *BhbZIP58* was likewise engaged in the ABA signaling pathway. In addition, BhbZIP58 had transcriptional activation activity and was localized in the nucleus, which suggest that BhbZIP58 acts as a transcriptional activator to regulate gene expression in response to stress (Fig. 8A,B). The function of *BhbZIP58* was preliminarily determined by overexpression in Arabidopsis under heat stress, indicating it could improve the ability of wax gourd to resist heat stress (Fig. 9). This result was similar to that of *AtbZIP17*, *EcbZIP17* and *OsbZIP60* in improving the heat tolerance of plants [21, 28, 29]. However, the regulatory mechanism of *BhbZIP58* in response to heat stress requires further experimental verification.

## Conclusions

In the present study, 61 *BhbZIP* genes were identified and divided into 12 subgroups by the bioinformatics method. We corrected and confirmed the CDS and gene structure of each *BhbZIP* gene using transcripts to ensure the accuracy of the results. It was worth noting that whole-genome duplication (WGD) and dispersed duplication (DSD) were the main driving forces of *bZIP* gene family expansion in wax gourd. *BhbZIP* gene might have undergone intense purifying selection pressure during the evolutionary process. In addition, *BhbZIP58* could rapidly respond to high temperature and its expression level was the highest in the root. BhbZIP58 located in the nucleus and had transcriptional activation activity, and that its overexpression improves heat stress tolerance in Arabidopsis. This study established the foundation for further research into the regulatory mechanism of *BhbZIP58* under heat stress in wax gourd, and promoting the molecular breeding of wax gourds.

## Materials and methods

### Identification of *BhbZIP* genes

We download the genome annotation and the genome sequences file of wax gourd from the Cucurbit Genomics Database (CuGenDB) (http://cucurbitgenomics.org/v2/) [45]. The Hidden Markov Model (HMM) configuration files of PF00170, PF07716, and PF03131 were downloaded from Pfam (https://pfam.xfam.org/) [46]. The raw bZIP proteins were found in the wax gourd genome using the hmmsearch function of the HMMER software (version 3.3), and multiple sequence alignment was carried out using clustalw (version 2.1) [47, 48]. Next, the hmmbuild function was used to build a wax gourd-specific *bZIP* gene family HMM model, and the E-value threshold was set to e^−5^. Using the updated HMM file, the members of *bZIP* gene family were once more identified from the wax gourd genome [49]. Candidate proteins that lacked the bZIP conserved domain or were severely deleted were excluded from further analysis using InterPro (version 91.0) and the National Center for Biotechnology Information (NCBI) Batch CD-Search Tools (https://www.NCBI.nlm.nih.gov/structure/bwrpsb/bwrpsb.cgi). To ensure gene accuracy, we also used the IGV-sRNA software (https://gitee.com/CJchen/IGV-sRNA) to correct and confirm the genes. Finally, we used ClustalX (version 2.1) and WebLogo (http://weblogo.threeplusone.com/create.cgi) for multiple sequence alignment and visualization, respectively [47, 50].

### Analysis of physicochemical properties of proteins

Isoelectric point (PIs), molecular weight (MWs), mean hydrophilicity, and charge were all examined using the ExPASy-ProtParam website (https://web.expasy.org/protparam/) [51]. Wax gourd genome annotation file was used to map the chromosome positions of family members through TBtools software (version 1.104) [52]. The online software Plant-mPLoc (http://www.csbio.sjtu.edu.cn/bioinf/plant-multi/) was utilized to predict the subcellular localization of BhbZIPs by uploading protein sequences [53]. Additionally, the secondary structure of BhbZIPs was predicted through the online software SOPMA (https://npsa-prabi.ibcp.fr/cgi-bin/npsa_automat.pl?page=/NPSA/npsa_sopma.html) [54].

### Conserved domain and phylogenetic tree analysis

All bZIP proteins sequence in Arabidopsis could be accessed from the TARE website (https://www.arabidopsis.org/). Pfam website (http://pfam.xfam.org/) was used to identify the position of conserved domain of BhbZIP and AtbZIP protein [46]. The following three steps should be taken to create a phylogenetic tree between *AtbZIP* and *BhbZIP*. First, the sequence of all bZIP protein will be aligned using mafft software [55]. Second, the gapthreshold value was set to 0.3 while trimming the multi-sequence alignment file using Trimal software [56]. The final step was to use IQ-TREE software to find the optimal model and build a maximum likelihood tree, and set the alert value to 1000 [57]. Then ITOL website (https://itol.embl.de/, version 6.6) was utilized to beautify the phylogenetic tree [58].

### Analysis of protein conserved motif and Gene structure

MEME (http://meme-suite.org/) website was used to identify the conserved motif of BhbZIP proteins [59]. The maximum motif parameter of the protein was set to 20, the rest of the parameters remain unchanged [9]. Gene structure information of *BhbZIP* was obtained from genome annotation files. The IGV-sRNA was then used to correct and confirm the gene structure of each *BhbZIP* including UTR regions, exons and introns by bam files of the transcriptome data. Then a phylogenetic tree was created specifically for only BhbZIPs using the method described above. Finally, visualization of the phylogenetic tree, conserved motifs, and gene structure were performed by ggplot2 (version 3.4.0) and ggtree (versionv3.6.1) [60, 61].

### Collinearity and gene duplication analysis of the *BhbZIPs*

The genome sequence and annotation data of rice, Arabidopsis, cucumber and grape were obtained from the Ensembl Plants website (http://plants.ensembl.org/index.html). BLASTP (version 2.13.0) and MCsanx software were then used to examine the inter-species collinearity relationship between wax gourd and rice, cucumber, grape, and Arabidopsis, and the intra-species collinearity relationship of wax gourd [62]. MCscanx and DupGen_finder attempts to identify the different modes of duplicated gene pairs in wax gourd by using grape as an outgroup [62, 63]. The synonymous and non-synonymous substitution (Ka/Ks) of the collinear gene pairs were determined using the ParaAT and KaKs_Calculator software [64, 65]. GC content and gene density of wax gourd genome were extracted by bedtools, and R program circlize was used for visualization [66].

### Cis-regulatory elements analysis and prediction of the interaction network

The 2000-bp DNA sequence upstream of the start codon was extracted from each wax gourd *bZIP* gene using the Tbtools software (version 1.104) [52]. Then, we analyzed Cis-regulatory Elements using the online tool PlantCRAE (http://bioinformatics.psb.ugent.be/webtools/plantcare/html/) and categorize Cis-regulatory Elements. We kept only the promoters on the positive chain and eliminated a very small number of promoters, which were visualized with ggplot2 [61]. STRING software (https://string-db.org/) was used to analyze the interaction of BhbZIP proteins (orthologs in Arabidopsis), and the confidence parameter was set to 0.4 [67].

### Plant material and stress treatments

The wax gourd seeds were provided by Vegetable Research Institute, Guangdong Academy of Agricultural Sciences [30]. The seeds were germinated and then sown in pots which were placed in an artificial climate chamber at 28 °C and 14/10 h for light /dark cycles. The same-growing and one-month-old seedlings were chosen and placed in the incubators at 42℃ and 8℃ for heat and cold stress treatment, respectively [68, 69]. We used 10% PEG6000 to simulate drought treatment and 9 g/L NaCl to simulate salt stress, both of which were treated with irrigated roots [70]. For ABA treatment, wax gourd leaves were sprayed with 0.2 g/L ABA [69]. At 0 h, 1 h, 3 h, 6 h, 12 h, and 24 h, the wax gourd leaves were collected, accordingly. The various wax gourd tissues were obtained from plants at the reproductive stage under normal growing circumstances. Three biological replicates were performed for all treatments, and samples were submerged in liquid nitrogen and then moved to a refrigerator at -80℃.

### Total RNA extraction and RT-qPCR

Total RNAs were extracted from different samples using a TransZol Up Plus RNA Kit (TransGen Biotech, Beijing, China) by the specification. Then the RNAs were reverse transcribed into the first-strand cDNA by a FastKing gDNA Dispelling RT SuperMix Kit (Tiangen Biotech, Beijing, China). *BhbZIP58 was* chosen for research using Real-time quantitative PCR (qPCR) to conduct the expression profile under abiotic stresses. Three replicates were conducted on a CFX96 Touch™ Real-Time PCR detection system (BIO-RAD, USA). Actin gene (*Bhi10M001911*) of wax gourd was used as a reference for normalization, whose primers were listed in Table S7 [71]. PCR reactions were performed using iTransStart® Green qPCR SuperMix (TransGen Biotech, Beijing, China). The relative mRNA expression level for each gene was calculated by the 2^−ΔΔCT^ method [72]. Gene-specific DNA primers for qPCR were presented in Table S7. To find the expression profiles with significant differences, the mean Ct values were statistically examined using Duncan’s test. *P* ≤ 0.05 was regarded as statistically significant.

### Subcellular localization analysis in tobacco

*BhbZIP58* gene was cloned in two separate segments (F1-R1, F2-R2) and then the total sequence was cloned using F1 and R2 primers (Table S7). The full-length coding sequence (CDS), without the stop codon of *BhbZIP58* was cloned and inserted into the pSuper-1300 GFP vector to fuse with the GFP tag. Then using the freeze–thaw method, the recombinant plasmid produced and empty vector were respectively introduced into *Agrobacterium tumefaciens* strain GV1301. The tobacco was grown in a greenhouse under the 16-h light/8-h dark cycle at 25℃ for five weeks. Agrobacterium was then incubated overnight and the bacteria were collected by centrifugal separation, diluted to an OD600 of 1 with a resuspension and injected into tobacco leaves. The infected plants were cultured for two days as normal after being kept in the dark for 24 h. Near the injection site of the leaves, the confocal laser scanning microscope (Zeiss LSM710, Jena, Germany) was utilized to observe the GFP fluorescence. Primers used for vector construction were listed in Table S7.

### Transcriptional activation activity analysis of *BhbZIP58*

pEASY®-Basic Seamless Cloning and Assembly Kit (TransGen Biotech, China) and *Nde*I and *Bam*HI restriction endonucleases (New England Biolabs, USA) were used to insert the CDS of *BhbZIP58* into the pGBKT7 vector. On a medium devoid of tryptophan (Trp) and histidine (His), pGBT9 was applied as a positive control (PC) that could activate the *HIS3*, *ADE2*, and *MEL1* reporter genes as well as α-galactosidase activity in the transformed yeast cells. pGBKT7 served as a negative control (NC). The yeast strain AH109 was transformed with the NC, PC, and pGBKT7-*BhbZIP58* plasmids, which were then cultivated on synthetic-defined (SD)/-Trp or SD/-Trp/-His medium with or without X-α-Gal.

### Heterologous overexpression in Arabidopsis

The CDS of BhbZIP58 was inserted into the pBI121 vector and controlled by the 35S promoter. A.tumefaciens strain GV3101 was transformed with the recombinant construct. Arabidopsis Columbia ecotype (Col-0) was used as the WT, which provided by Vegetable Research Institute, Guangdong Academy of Agricultural Sciences. The Arabidopsis plants were grown in a growth room at 22℃ with a 16-h light/8-h dark cycle. Agrobacterium-mediated transformation of Arabidopsis was performed using the floral dip method [73]. Transgenic plants were identified by kanamycin resistance and further confirmed by qPCR. Arabidopsis T3-generation plants were used for functional identification. For the seedling survival assays, we planted 20 seeds of WT and three overexpression lines. The control group was grown at 22 °C for 12 days, while the treatment group underwent the following heat stress conditions: incubation at 22 °C for 5 days, followed by exposure to 37 °C for 90 min, then returning to 22 °C for 90 min, subsequently subjecting the seedlings to 44 °C for 90 min, and finally returning at 22 °C for 7 days [74]. Three biological replicates were performed for all treatments. The survival rate of the 20 Arabidopsis seedlings was then used to represent the transgenic lines.

### Supplementary Information

**Additional file 1.** **Fig. S1.** Visualization of multiple sequence alignment of bZIP domain in wax gourd. The overall height from the letter piles at each point shows the sequence conservation at that position (measured in bits). The relative frequency of the corresponding amino acid at each position was represented by the height of a single letter in the letter stacks. **Fig. S2.** 20 motifs of *BhbZIP* genes. The height of the letters indicates the conserved amino acids, with different bases in different colors. **Fig. S3.** Synteny analysis of the bZIP genes between wax gourd, and (A) Arabidopsis, (B) Cucumber, (C) Rice, (D) Grapes. The different colour lines indicated gene blocks in wax gourd that were orthologous to the other genomes, which delineate the collinear bZIP gene pairs. (E)The numbers of bZIP genes forming syntenic pairs between wax gourd and other four species which visualized using Venn plot. **Fig. S4.** Relative expression levels of different subgroups of *BhbZIP* under heat stress. Data represents the average of three biological replicates, with error bars indicating standard deviation. Single-factor analysis of variance (ANOVA) was performed using Duncan's test to assess the expression levels of *BhbZIP* at 0, 1, 3, 6, 12, and 24 hours. Different letters denote significant differences at the 0.05 level. **Fig. S5.** Relative expression levels of *BhbZIP58* and *LHY(Bhi12G002045) *under normal growth conditions. Data represents the average of three biological replicates, with error bars indicating standard deviation. Single-factor analysis of variance (ANOVA) was performed using Duncan's test to assess the expression levels of *BhbZIP* at 0, 1, 3, 6, 12, and 24 hours. Different letters denote significant differences at the 0.05 level. **Fig. S6.** Alignment of BhbZIP58 with other Cucurbitaceae homologous proteins. The letters at the bottom indicate the conservatism of the base and the overall height from the letter piles at each point shows the sequence conservation at that position. **Fig. S7.** Clone of BhbZIP58. Marker, Trans 8000bp DNA Marker. **Fig. S8.** Expression of *BhbZIP58* in WT and 35S:*BhbZIP58* seedlings. Values are means ± SDs (*n* = 3); **, *p* < 0.01 (Student’s t-test). **Table S1.** Basic information of the *bZIP* genes identified in wax gourd. **Table S2.** Secondary structure and subcellular localization of the 61 *BhbZIP* gene family members. **Table S3.** Protein–Protein Interaction of online STRING. **Table S4.** Gene duplication type of the *BhbZIP* gene family.**Table S5.** Selective pressure analysis of the *BhbZIP* gene family. **Table S6.** Collinear *bZIP* gene pairs between species. **Table S7.** Primers used in this study.

## Data Availability

All the relevant data are included within the article and its additional files.

## Abbreviations

bzip: Basic Leucine Zipper
pI: Theoretical Isoelectric Point
Mw: Molecular Weight
MEME: Multiple Expectation Maximization for Motif Elicitation
MCScanX: Multiple Collinearity Scan toolkit
Ka: Non-synonymous
Ks: Synonymous
ML: Maximum Likelihood
cDNA: Complementary DNA
qRT-PCR: Quantitative real-time polymerase chain reaction

## References

[1] Rushton PJ, Somssich IE, Ringler P, Shen QJ (2010). WRKY transcription factors. Trends Plant Sci.

[2] Singh KB, Foley RC, Oñate-Sánchez L (2002). Transcription factors in plant defense and stress responses. Curr Opin Plant Biol.

[3] Dubos C, Stracke R, Grotewold E, Weisshaar B, Martin C, Lepiniec L (2010). MYB transcription factors in Arabidopsis. Trends Plant Sci.

[4] Mitsuda N, Ohme-Takagi M (2009). Functional analysis of transcription factors in Arabidopsis. Plant Cell Physiol.

[5] Taylor-Teeples M, Lin L, de Lucas M, Turco G, Toal TW, Gaudinier A (2015). An Arabidopsis gene regulatory network for secondary cell wall synthesis. Nature.

[6] Das Gupta M, Tsiantis M (2018). Gene networks and the evolution of plant morphology. Curr Opin Plant Biol.

[7] Feller A, Machemer K, Braun EL, Grotewold E (2011). Evolutionary and comparative analysis of MYB and bHLH plant transcription factors. Plant J.

[8] Jakoby M, Weisshaar B, Dröge-Laser W, Vicente-Carbajosa J, Tiedemann J, Kroj T (2002). bZIP transcription factors in Arabidopsis. Trends Plant Sci.

[9] Dröge-Laser W, Snoek BL, Snel B, Weiste C (2018). The Arabidopsis bZIP transcription factor family — an update. Curr Opin Plant Biol.

[10] Hurst HC (1995). Transcription factors 1: bZIP proteins. Protein Profile.

[11] Izawa T, Foster R, Chua NH (1993). Plant bZIP protein DNA binding specificity. J Mol Biol.

[12] Wei K, Chen J, Wang Y, Chen Y, Chen S, Lin Y (2012). Genome-wide analysis of bZIP-encoding genes in maize. DNA Res.

[13] E ZG, Zhang YP, Zhou JH, Wang L. Mini review roles of the bZIP gene family in rice. Genet Mol Res. 2014;13:3025–36. 10.4238/2014.April.16.11 24782137

[14] Baloglu MC, Eldem V, Hajyzadeh M, Unver T (2014). Genome-wide analysis of the bZIP transcription factors in cucumber. PLoS ONE.

[15] Liu J, Chen N, Chen F, Cai B, Dal Santo S, Tornielli GB (2014). Genome-wide analysis and expression profile of the bZIP transcription factor gene family in grapevine (*Vitis vinifera*). BMC Genomics.

[16] Zhao K, Chen S, Yao W, Cheng Z, Zhou B, Jiang T (2021). Genome-wide analysis and expression profile of the bZIP gene family in poplar. BMC Plant Biol.

[17] Li D, Fu F, Zhang H, Song F (2015). Genome-wide systematic characterization of the bZIP transcriptional factor family in tomato (*Solanum lycopersicum* L.). BMC Genomics.

[18] Wang Q, Guo C, Li Z, Sun J, Wang D, Xu L (2021). Identification and analysis of bZIP family genes in potato and their potential roles in stress responses. Front Plant Sci.

[19] Manghwar H, Li J (2022). Endoplasmic reticulum stress and unfolded protein response signaling in plants. Int J Mol Sci.

[20] Srivastava R, Deng Y, Howell SH (2014). Stress sensing in plants by an ER stress sensor/transducer, bZIP28. Front Plant Sci.

[21] Liu J-X, Srivastava R, Howell SH (2008). Stress-induced expression of an activated form of AtbZIP17 provides protection from salt stress in Arabidopsis. Plant, Cell Environ.

[22] Fujita M, Mizukado S, Fujita Y, Ichikawa T, Nakazawa M, Seki M (2007). Identification of stress-tolerance-related transcription-factor genes via mini-scale full-length cDNA Over-eXpressor (FOX) gene hunting system. Biochem Biophys Res Commun.

[23] Gao H, Brandizzi F, Benning C, Larkin RM (2008). A membrane-tethered transcription factor defines a branch of the heat stress response in *Arabidopsis thaliana*. Proc Natl Acad Sci U S A.

[24] Liu J-X, Srivastava R, Che P, Howell SH (2007). Salt stress responses in Arabidopsis utilize a signal transduction pathway related to endoplasmic reticulum stress signaling: Salt stress elicits ER stress response. Plant J.

[25] Kim J-S, Yamaguchi-Shinozaki K, Shinozaki K (2018). ER-Anchored transcription factors bZIP17 and bZIP28 regulate root elongation. Plant Physiol.

[26] Gao J, Wang M-J, Wang J-J, Lu H-P, Liu J-X (2022). bZIP17 regulates heat stress tolerance at reproductive stage in Arabidopsis. aBIOTECH.

[27] Geng X, Zang X, Wang F, Zhang L, Tian X, Ni Z (2016). Isolation and function analysis of heat stress related transcription factor gene TabZIP28 in wheat (*Triticum aestivum*). J Agri Biotechnol.

[28] Yu X, Niu X, Yang S, Li Y, Liu L, Tang W (2011). Research on heat and drought tolerance in rice (*Oryza sativa* L.) by overexpressing transcription factor OsbZIP60. Scientia Agricultura Sinica.

[29] Ramakrishna C, Singh S, Raghavendrarao S, Padaria JC, Mohanty S, Sharma TR (2018). The membrane tethered transcription factor EcbZIP17 from finger millet promotes plant growth and enhances tolerance to abiotic stresses. Sci Rep.

[30] Yan J, Wang M, Liu W, Xie D, He X, Peng Q (2021). Identification and characterization of known and novel MicroRNAs in five tissues of wax gourd (*Benincasa hispida*) based on high-throughput sequencing. Appl Sci.

[31] Ma L, Liu Z, Cheng Z, Gou J, Chen J, Yu W (2021). Identification and application of BhAPRR2 controlling peel colour in wax gourd (*Benincasa hispida*). Front Plant Sci.

[32] Grover JK, Adiga G, Vats V, Rathi SS (2001). Extracts of *Benincasa hispida* prevent development of experimental ulcers. J Ethnopharmacol.

[33] Gu M, Fan S, Liu G, Guo L, Ding X, Lu Y (2013). Extract of Wax gourd peel prevents high-fat diet-induced hyperlipidemia in C57BL/6 mice via the inhibition of the PPARγ pathway. Evid Based Complement Alternat Med.

[34] Fahad S, Bajwa AA, Nazir U, Anjum SA, Farooq A, Zohaib A (2017). Crop production under drought and heat stress: plant responses and management options. Front Plant Sci.

[35] Wang M, Jiang B, Liu W, Lin Y, Liang Z, He X (2019). Transcriptome Analyses Provide Novel Insights into Heat Stress Responses in Chieh-Qua (*Benincasa hispida* Cogn. var. *Chieh-Qua* How). Int J Mol Sci.

[36] Xie D, Xu Y, Wang J, Liu W, Zhou Q, Luo S (2019). The wax gourd genomes offer insights into the genetic diversity and ancestral cucurbit karyotype. Nat Commun.

[37] Holub EB (2001). The arms race is ancient history in Arabidopsis, the wildflower. Nat Rev Genet.

[38] Yang Y, Li J, Li H, Yang Y, Guang Y, Zhou Y (2019). The bZIP gene family in watermelon: genome-wide identification and expression analysis under cold stress and root-knot nematode infection. PeerJ.

[39] Wang S, Zhang X, Li B, Zhao X, Shen Y, Yuan Z (2022). Genome-wide identification and characterization of bZIP gene family and cloning of candidate genes for anthocyanin biosynthesis in pomegranate (*Punica granatum*). BMC Plant Biol.

[40] Ma M, Chen Q, Dong H, Zhang S, Huang X (2021). Genome-wide identification and expression analysis of the bZIP transcription factors, and functional analysis in response to drought and cold stresses in pear (*Pyrus breschneideri*). BMC Plant Biol.

[41] Cannon SB, Mitra A, Baumgarten A, Young ND, May G (2004). The roles of segmental and tandem gene duplication in the evolution of large gene families in *Arabidopsis thaliana*. BMC Plant Biol.

[42] He Q, Cai H, Bai M, Zhang M, Chen F, Huang Y (2020). A soybean bZIP transcription factor GmbZIP19 Confers multiple biotic and abiotic stress responses in plant. IJMS.

[43] Tang H, Bowers JE, Wang X, Ming R, Alam M, Paterson AH (2008). Synteny and collinearity in plant genomes. Science.

[44] Abe H, Yamaguchi-Shinozaki K, Urao T, Iwasaki T, Hosokawa D, Shinozaki K (1997). Role of arabidopsis MYC and MYB homologs in drought- and abscisic acid-regulated gene expression. Plant Cell.

[45] Yu J, Wu S, Sun H, Wang X, Tang X, Guo S, et al. CuGenDBv2: an updated database for cucurbit genomics. Nucleic Acids Res. 2022;:gkac921. 10.1093/nar/gkac921 PMC9825510 36271794

[46] Mistry J, Chuguransky S, Williams L, Qureshi M, Salazar GA, Sonnhammer ELL (2020). Pfam: the protein families database in 2021. Nucleic Acids Res.

[47] Larkin MA, Blackshields G, Brown NP, Chenna R, McGettigan PA, McWilliam H (2007). Clustal W and Clustal X version 2.0. Bioinformatics.

[48] Finn RD, Clements J, Eddy SR. HMMER web server: interactive sequence similarity searching. Nucleic Acids Res. 2011;39 Web Server issue:W29–37. 10.1093/nar/gkr367 PMC3125773 21593126

[49] Lozano R, Hamblin MT, Prochnik S, Jannink J-L (2015). Identification and distribution of the NBS-LRR gene family in the Cassava genome. BMC Genomics.

[50] Crooks GE, Hon G, Chandonia J-M, Brenner SE (2004). WebLogo: a sequence logo generator. Genome Res.

[51] Artimo P, Jonnalagedda M, Arnold K, Baratin D, Csardi G, de Castro E, et al. ExPASy: SIB bioinformatics resource portal. Nucleic Acids Res. 2012;40 Web Server issue:W597–603. 10.1093/nar/gks400 PMC3394269 22661580

[52] Chen C, Chen H, Zhang Y, Thomas HR, Frank MH, He Y (2020). TBtools: an integrative toolkit developed for interactive analyses of big biological data. Mol Plant.

[53] Chou K-C, Shen H-B (2010). Plant-mPLoc: a top-down strategy to augment the power for predicting plant protein subcellular localization. PLoS ONE.

[54] Geourjon C, Deléage G (1995). SOPMA: significant improvements in protein secondary structure prediction by consensus prediction from multiple alignments. Comput Appl Biosci.

[55] Nakamura T, Yamada KD, Tomii K, Katoh K (2018). Parallelization of MAFFT for large-scale multiple sequence alignments. Bioinformatics.

[56] Capella-Gutiérrez S, Silla-Martínez JM, Gabaldón T (2009). trimAl: a tool for automated alignment trimming in large-scale phylogenetic analyses. Bioinformatics.

[57] Minh BQ, Schmidt HA, Chernomor O, Schrempf D, Woodhams MD, von Haeseler A (2020). IQ-TREE 2: new models and efficient methods for phylogenetic inference in the genomic era. Mol Biol Evol.

[58] Letunic I, Bork P (2021). Interactive tree of life (iTOL) v5: an online tool for phylogenetic tree display and annotation. Nucleic Acids Res.

[59] Bailey TL, Williams N, Misleh C, Li WW. MEME: discovering and analyzing DNA and protein sequence motifs. Nucleic Acids Res. 2006;34 Web Server issue:W369–373. 10.1093/nar/gkl198 PMC1538909 16845028

[60] Yu G (2020). Using ggtree to visualize data on tree-like structures. Curr Protoc Bioinformatics.

[61] Villanueva RAM, Chen ZJ. ggplot2: Elegant Graphics for Data Analysis (2nd ed.). Measurement: Interdisciplinary Research and Perspectives. 2019;17:160–7.

[62] Wang Y, Tang H, DeBarry JD, Tan X, Li J, Wang X (2012). MCScanX: a toolkit for detection and evolutionary analysis of gene synteny and collinearity. Nucleic Acids Res.

[63] Qiao X, Li Q, Yin H, Qi K, Li L, Wang R (2019). Gene duplication and evolution in recurring polyploidization-diploidization cycles in plants. Genome Biol.

[64] Zhang Z, Xiao J, Wu J, Zhang H, Liu G, Wang X (2012). ParaAT: a parallel tool for constructing multiple protein-coding DNA alignments. Biochem Biophys Res Commun.

[65] Wang D, Zhang Y, Zhang Z, Zhu J, Yu J (2010). KaKs_Calculator 2.0: a toolkit incorporating gamma-series methods and sliding window strategies. Genomics Proteomics Bioinformatics.

[66] Gu Z, Gu L, Eils R, Schlesner M, Brors B (2014). circlize Implements and enhances circular visualization in R. Bioinformatics.

[67] Szklarczyk D, Gable AL, Nastou KC, Lyon D, Kirsch R, Pyysalo S (2021). The STRING database in 2021: customizable protein-protein networks, and functional characterization of user-uploaded gene/measurement sets. Nucleic Acids Res.

[68] Dong C-J, Li L, Shang Q-M, Liu X-Y, Zhang Z-G (2014). Endogenous salicylic acid accumulation is required for chilling tolerance in cucumber (*Cucumis sativus* L.) seedlings. Planta.

[69] Song Q, Li D, Dai Y, Liu S, Huang L, Hong Y (2015). Characterization, expression patterns and functional analysis of the MAPK and MAPKK genes in watermelon (*Citrullus lanatus*). BMC Plant Biol.

[70] Li Y, Li S, He X, Jiang W, Zhang D, Liu B (2020). CO2 enrichment enhanced drought resistance by regulating growth, hydraulic conductivity and phytohormone contents in the root of cucumber seedlings. Plant Physiol Biochem.

[71] Cheng Z, Liu Z, Xu Y, Ma L, Chen J, Gou J (2021). Fine mapping and identification of the candidate gene BFS for fruit shape in wax gourd (*Benincasa hispida*). Theor Appl Genet.

[72] Livak KJ, Schmittgen TD (2001). Analysis of relative gene expression data using real-time quantitative PCR and the 2(-Delta Delta C(T)) Method. Methods.

[73] Clough SJ, Bent AF (1998). Floral dip: a simplified method for Agrobacterium-mediated transformation of *Arabidopsis thaliana*: Floral dip transformation of Arabidopsis. Plant J.

[74] Sedaghatmehr M, Mueller-Roeber B, Balazadeh S (2016). The plastid metalloprotease FtsH6 and small heat shock protein HSP21 jointly regulate thermomemory in Arabidopsis. Nat Commun.

