# Supplementary material for: Genome-wide identification of bZIP gene family and expression analysis of BhbZIP58 under heat stress in wax gourd
Source: BMC Plant Biol. 2023 Nov 29;23:598. doi: 10.1186/s12870-023-04580-6 (PMC10685590; doi:10.1186/s12870-023-04580-6)
Supplement: Supplementary file 1 — Additional file 1. Fig. S1. Visualization of multiple sequence alignment of bZIP domain in wax gourd. The overall height from the letter piles at each point shows the sequence conservation at that position (measured in bits). The relative frequency of the corresponding amino acid at each position was represented by the height of a single letter in the letter stacks. Fig. S2. 20 motifs of BhbZIP genes. The height of the letters indicates the conserved amino acids, with different bases in different colors. Fig. S3. Synteny analysis of the bZIP genes between wax gourd, and (A) Arabidopsis, (B) Cucumber, (C) Rice, (D) Grapes. The different colour lines indicated gene blocks in wax gourd that were orthologous to the other genomes, which delineate the collinear bZIP gene pairs. (E)The numbers of bZIP genes forming syntenic pairs between wax gourd and other four species which visualized using Venn plot. Fig. S4. Relative expression levels of different subgroups of BhbZIP under heat stress. Data represents the average of three biological replicates, with error bars indicating standard deviation. Single-factor analysis of variance (ANOVA) was performed using Duncan's test to assess the expression levels of BhbZIP at 0, 1, 3, 6, 12, and 24 hours. Different letters denote significant differences at the 0.05 level. Fig. S5. Relative expression levels of BhbZIP58 and LHY(Bhi12G002045) under normal growth conditions. Data represents the average of three biological replicates, with error bars indicating standard deviation. Single-factor analysis of variance (ANOVA) was performed using Duncan's test to assess the expression levels of BhbZIP at 0, 1, 3, 6, 12, and 24 hours. Different letters denote significant differences at the 0.05 level. Fig. S6. Alignment of BhbZIP58 with other Cucurbitaceae homologous proteins. The letters at the bottom indicate the conservatism of the base and the overall height from the letter piles at each point shows the sequence conservation at that positi [file 12870_2023_4580_MOESM1_ESM.docx]

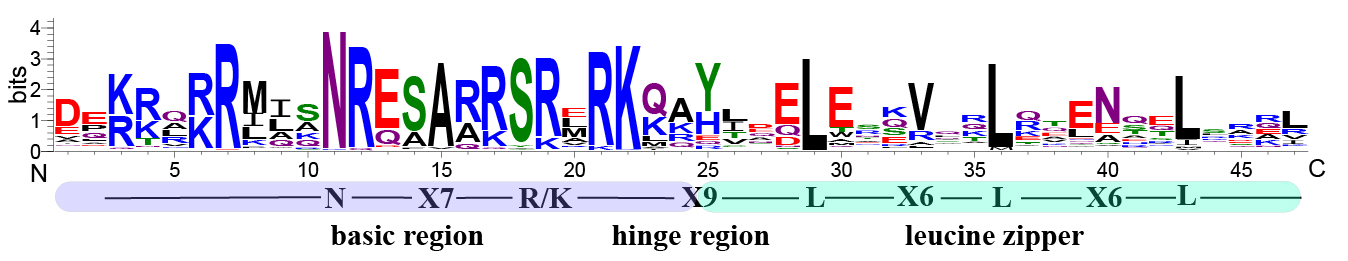


**Fig. S1** Visualization of multiple sequence alignment of bZIP domain in wax gourd. The overall height from the letter piles at each point shows the sequence conservation at that position (measured in bits). The relative frequency of the corresponding amino acid at each position was represented by the height of a single letter in the letter stacks.


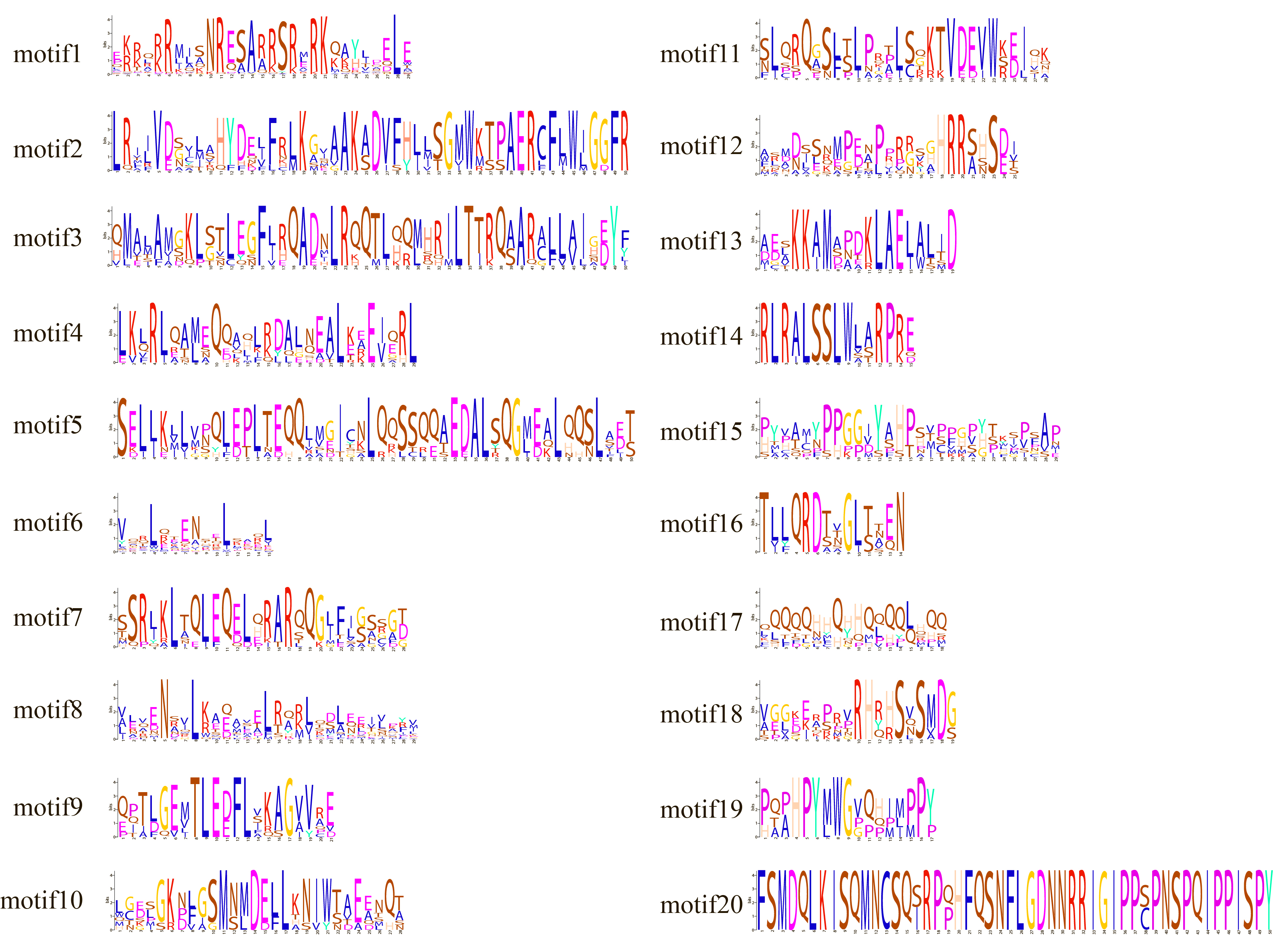


**Fig. S2** 20 motifs of *BhbZIP* genes. The height of the letters indicates the conserved amino acids, with different bases in different colors.


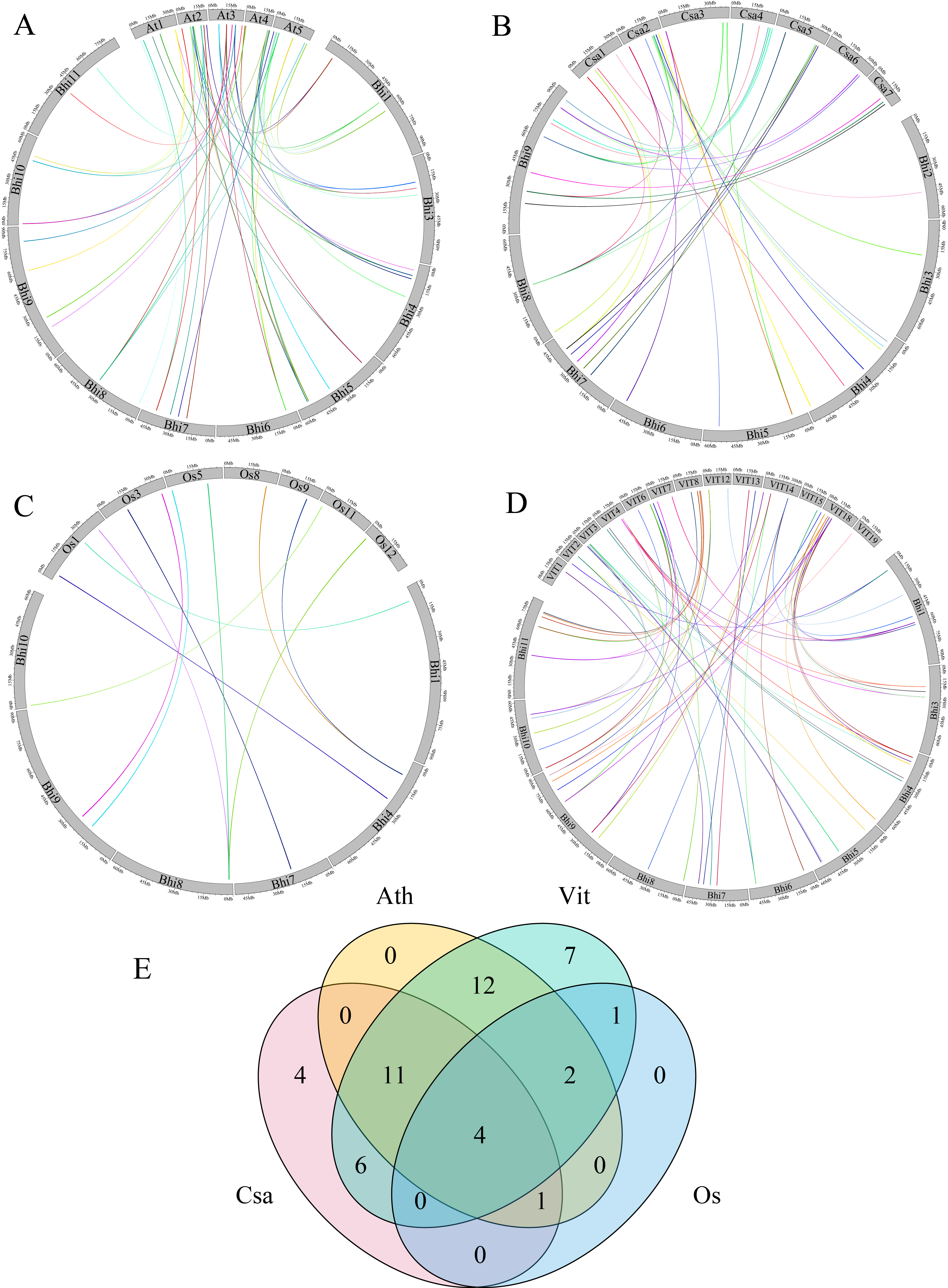


**Fig. S3** Synteny analysis of the bZIP genes between wax gourd, and (A) Arabidopsis, (B) Cucumber, (C) Rice, (D) Grapes. The different colour lines indicated gene blocks in wax gourd that were orthologous to the other genomes, which delineate the collinear bZIP gene pairs. (E)The numbers of bZIP genes forming syntenic pairs between wax gourd and other four species which visualized using Venn plot.


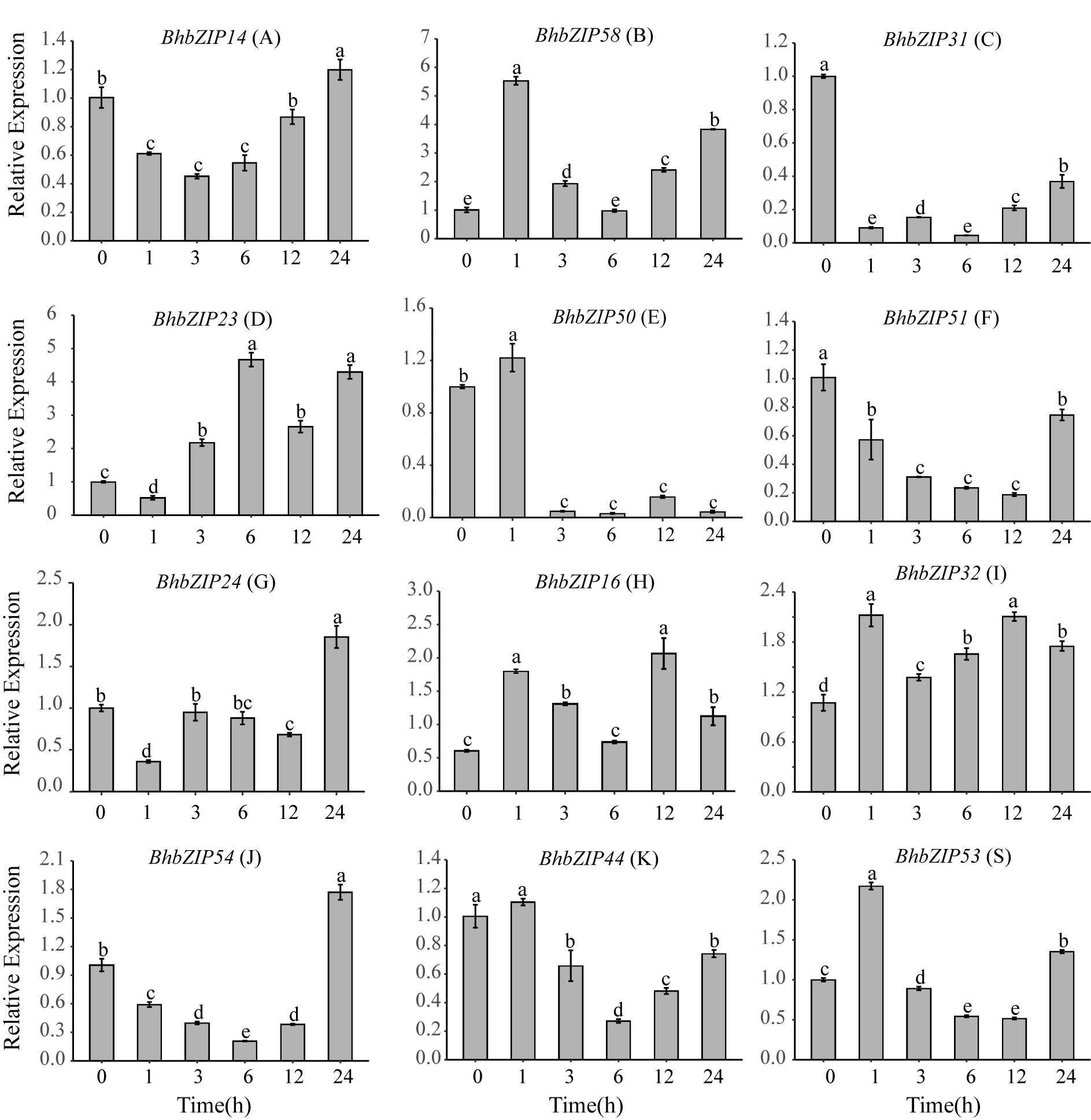


**Fig. S4** Relative expression levels of different subgroups of *BhbZIP* under heat stress. Data represents the average of three biological replicates, with error bars indicating standard deviation. Single-factor analysis of variance (ANOVA) was performed using Duncan's test to assess the expression levels of *BhbZIP* at 0, 1, 3, 6, 12, and 24 hours. Different letters denote significant differences at the 0.05 level.


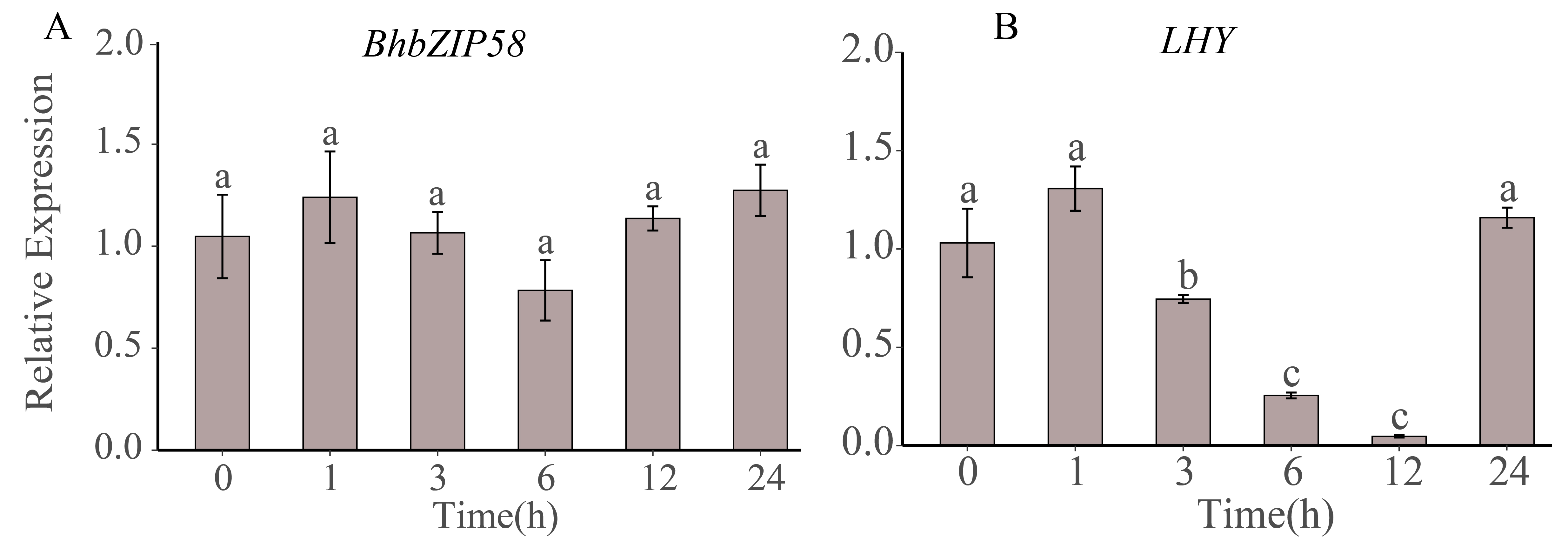


**Fig. S5** Relative expression levels of *BhbZIP58* and *LHY(Bhi12G002045)* under normal growth conditions. Data represents the average of three biological replicates, with error bars indicating standard deviation. Single-factor analysis of variance (ANOVA) was performed using Duncan's test to assess the expression levels of *BhbZIP* at 0, 1, 3, 6, 12, and 24 hours. Different letters denote significant differences at the 0.05 level.


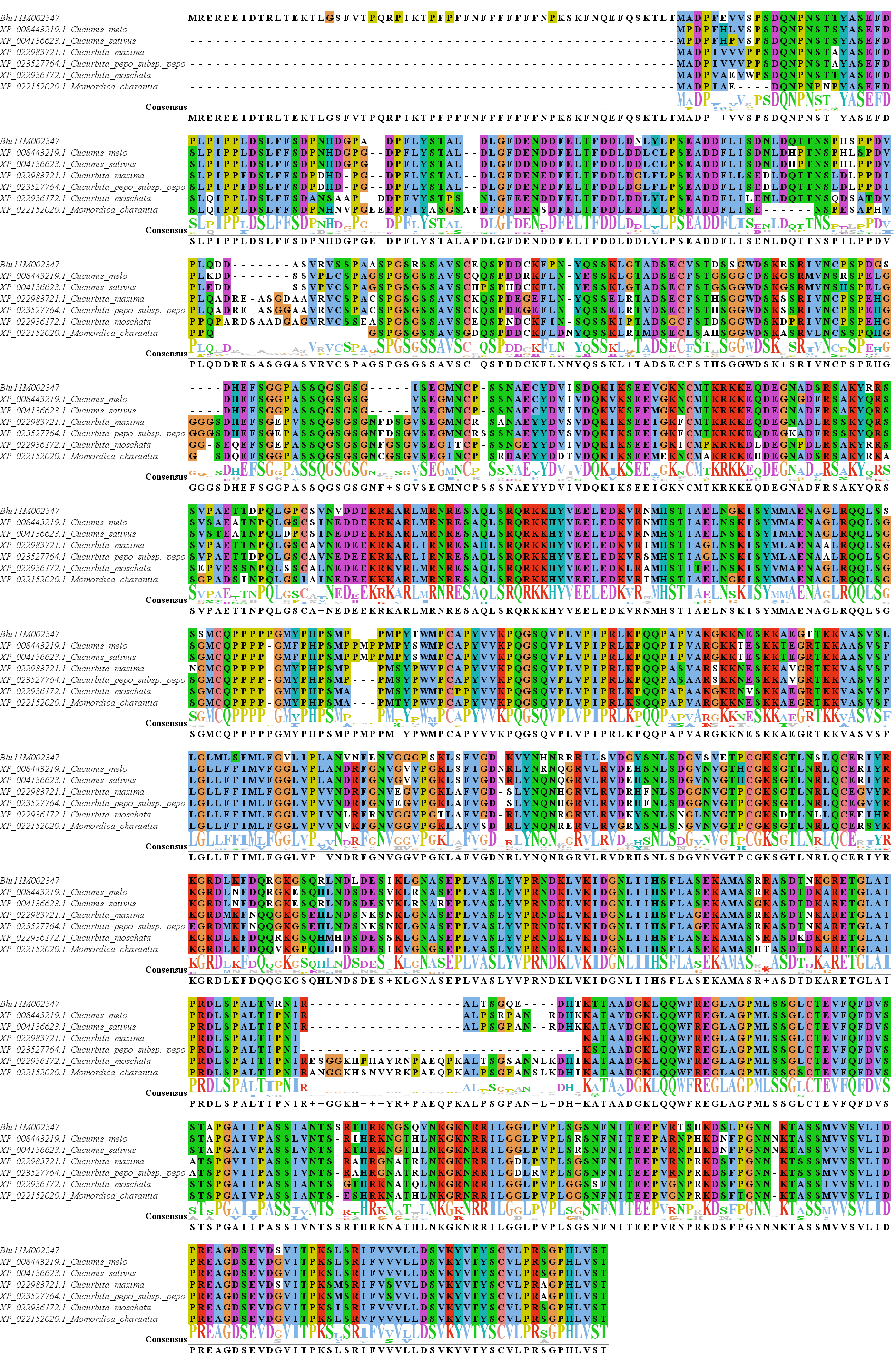


**Fig. S6** Alignment of BhbZIP58 with other Cucurbitaceae homologous proteins. The letters at the bottom indicate the conservatism of the base and the overall height from the letter piles at each point shows the sequence conservation at that position.

**
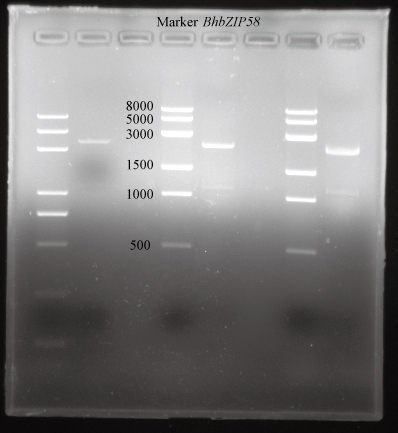
**

**Fig. S7** Clone of BhbZIP58. Marker, Trans 8000bp DNA Marker.


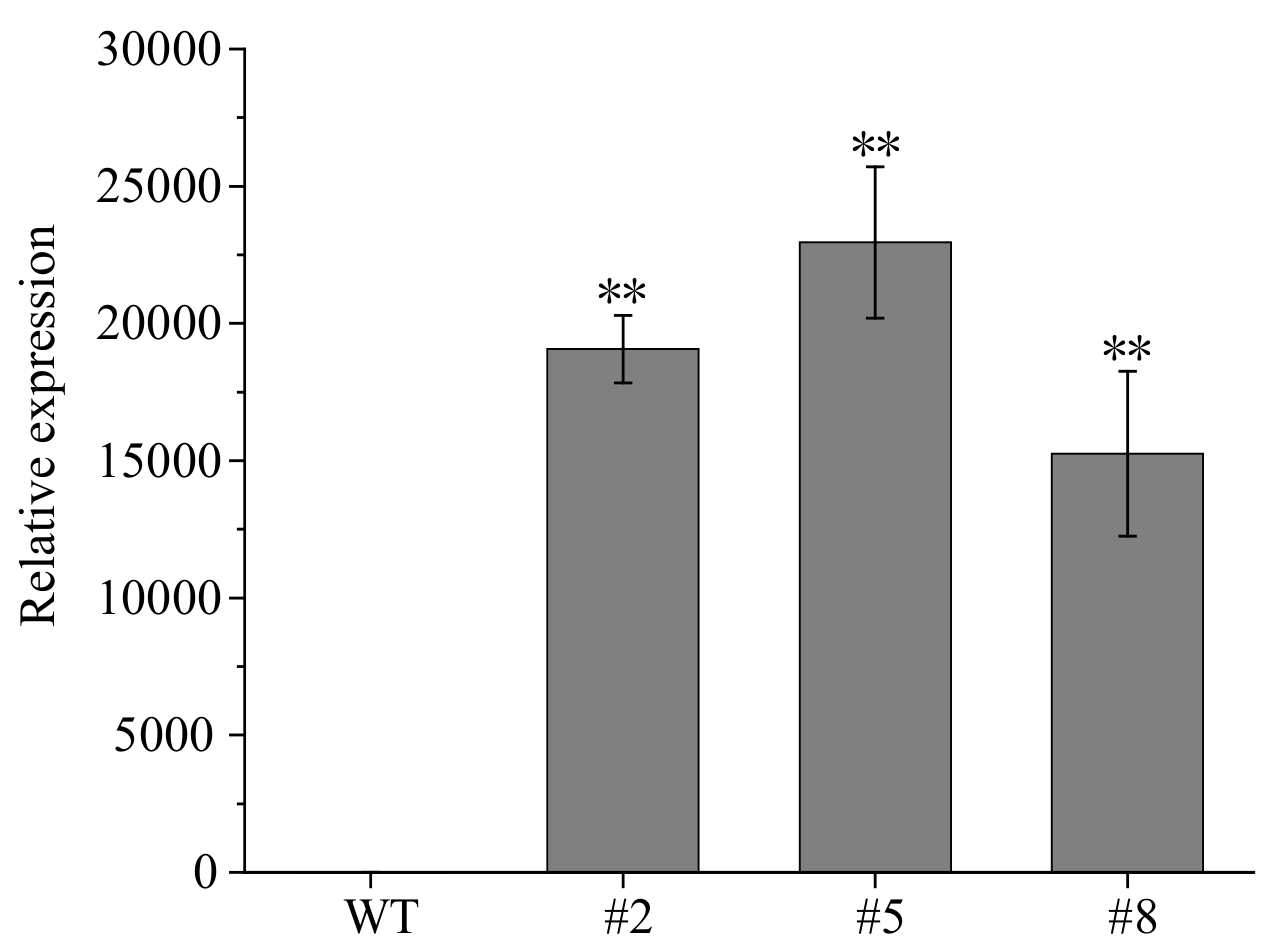


**Fig. S8** Expression of *BhbZIP58* in WT and 35S:*BhbZIP58* seedlings. Values are means ± SDs (n = 3); **, p < 0.01 (Student’s t-test).

**Table S1** Basic information of the *bZIP* genes identified in wax gourd

| Gene name | GeneID | ChrID | Start：end | Length(aa) | PI | MW(KDa) | GRAVY |
| --- | --- | --- | --- | --- | --- | --- | --- |
| BhbZIP1 | Bhi01G000318 | chr1 | 7530748:7536413 | 417 | 9.05 | 44.31 | -0.705 |
| BhbZIP2 | Bhi01G000868 | chr1 | 23273843:23291175 | 419 | 6.11 | 45.67 | -0.774 |
| BhbZIP3 | Bhi01G000872 | chr1 | 23411561:23422206 | 413 | 6.44 | 45.46 | -0.785 |
| BhbZIP4 | Bhi01G001186 | chr1 | 32661852:32668840 | 378 | 7.94 | 42.69 | -1.023 |
| BhbZIP5 | Bhi01G001706 | chr1 | 53011531:53013251 | 163 | 6.15 | 19.12 | -1.022 |
| BhbZIP6 | Bhi01G001873 | chr1 | 58550380:58551754 | 151 | 5.61 | 17.22 | -0.595 |
| BhbZIP7 | Bhi01G001913 | chr1 | 60375671:60383941 | 323 | 7.11 | 36.88 | -0.588 |
| BhbZIP8 | Bhi02G001384 | chr2 | 45029004:45036543 | 273 | 7.89 | 30.87 | -0.844 |
| BhbZIP9 | Bhi03G000687 | chr3 | 17267714:17270928 | 140 | 5.11 | 16.18 | -0.895 |
| BhbZIP10 | Bhi03G000814 | chr3 | 21508563:21510071 | 210 | 7.74 | 24.75 | -0.899 |
| BhbZIP11 | Bhi03G000984 | chr3 | 26322523:26329349 | 364 | 6.49 | 38.42 | -0.919 |
| BhbZIP12 | Bhi04G000003 | chr4 | 57745:65058 | 333 | 9.62 | 37.23 | -0.588 |
| BhbZIP13 | Bhi04G000176 | chr4 | 4884614:4885915 | 136 | 6.11 | 15.71 | -0.633 |
| BhbZIP14 | Bhi04G000330 | chr4 | 8835126:8839407 | 411 | 9.58 | 44.36 | -0.588 |
| BhbZIP15 | Bhi04G000415 | chr4 | 11263231:11264074 | 176 | 7.06 | 20.64 | -0.45 |
| BhbZIP16 | Bhi04G000795 | chr4 | 24471147:24473685 | 158 | 10.56 | 17.54 | -1.149 |
| BhbZIP17 | Bhi04G000896 | chr4 | 27409625:27413653 | 327 | 6.33 | 36.02 | -0.677 |
| BhbZIP18 | Bhi04G001290 | chr4 | 40810370:40816363 | 321 | 8.56 | 35.91 | -0.862 |
| BhbZIP19 | Bhi04G001359 | chr4 | 43011626:43015983 | 322 | 9.48 | 35.53 | -0.706 |
| BhbZIP20 | Bhi04G001371 | chr4 | 43255315:43257783 | 350 | 7.86 | 38.16 | -0.653 |
| BhbZIP21 | Bhi04G001577 | chr4 | 50855951:50859846 | 467 | 5.92 | 51.6 | -0.382 |
| BhbZIP22 | Bhi04G001617 | chr4 | 53721120:53721711 | 152 | 8.32 | 18.19 | -0.9 |
| BhbZIP23 | Bhi04G002048 | chr4 | 67789639:67795009 | 400 | 6.69 | 45.87 | -0.528 |
| BhbZIP24 | Bhi05G000290 | chr5 | 9230572:9238888 | 401 | 6.78 | 42.73 | -0.921 |
| BhbZIP25 | Bhi05G000555 | chr5 | 22477177:22483880 | 269 | 6.49 | 30.21 | -0.751 |
| BhbZIP26 | Bhi05G000928 | chr5 | 38558704:38563060 | 409 | 9.49 | 44.84 | -0.603 |
| BhbZIP27 | Bhi05G001473 | chr5 | 55542151:55545169 | 272 | 5.87 | 29.85 | -0.664 |
| BhbZIP28 | Bhi05G001531 | chr5 | 56537548:56541372 | 566 | 6 | 61.83 | -0.873 |
| BhbZIP29 | Bhi05G001533 | chr5 | 56550412:56554943 | 576 | 6.84 | 63.44 | -0.941 |
| BhbZIP30 | Bhi06G000246 | chr6 | 6798813:6800063 | 179 | 6.31 | 21.08 | -1.039 |
| BhbZIP31 | Bhi06G001517 | chr6 | 49889635:49894255 | 434 | 6.35 | 46.7 | -0.691 |
| BhbZIP32 | Bhi07G000420 | chr7 | 20197966:20204947 | 343 | 5.97 | 37.96 | -0.682 |
| BhbZIP33 | Bhi07G000619 | chr7 | 26236299:26243837 | 286 | 8.87 | 31.91 | -0.651 |
| BhbZIP34 | Bhi07G000824 | chr7 | 32032058:32039171 | 423 | 7.12 | 45.69 | -0.865 |
| BhbZIP35 | Bhi07G001014 | chr7 | 37901498:37908733 | 331 | 8.92 | 37.23 | -0.561 |
| BhbZIP36 | Bhi07G001174 | chr7 | 42289776:42291848 | 200 | 6.11 | 23.01 | -0.738 |
| BhbZIP37 | Bhi08G000051 | chr8 | 2022959:2027458 | 395 | 7.76 | 44.68 | -0.484 |
| BhbZIP38 | Bhi08G000880 | chr8 | 34406425:34407567 | 211 | 6.51 | 24.52 | -0.848 |
| BhbZIP39 | Bhi09G000681 | chr9 | 18482223:18483529 | 140 | 8.09 | 16.45 | -0.698 |
| BhbZIP40 | Bhi09G000917 | chr9 | 26060663:26084212 | 441 | 6.21 | 48.38 | -0.783 |
| BhbZIP41 | Bhi09G001219 | chr9 | 37927622:37931189 | 350 | 6.81 | 38.54 | -0.796 |
| BhbZIP42 | Bhi09G001870 | chr9 | 61450898:61452333 | 177 | 6.29 | 20.65 | -0.809 |
| BhbZIP43 | Bhi09G002211 | chr9 | 70900539:70902105 | 203 | 9.45 | 23.28 | -0.743 |
| BhbZIP44 | Bhi09G002310 | chr9 | 73484963:73488510 | 357 | 4.45 | 38.93 | -0.46 |
| BhbZIP45 | Bhi09G002631 | chr9 | 82194776:82195425 | 153 | 9.62 | 18.25 | -0.831 |
| BhbZIP46 | Bhi09G002846 | chr9 | 87934169:87937265 | 324 | 6.14 | 35.85 | -0.83 |
| BhbZIP47 | Bhi10G000025 | chr10 | 983201:985428 | 345 | 7.62 | 39.2 | -0.744 |
| BhbZIP48 | Bhi10G000031 | chr10 | 1156663:1165193 | 519 | 6.78 | 58.83 | -0.592 |
| BhbZIP49 | Bhi10G000691 | chr10 | 17973917:17977572 | 391 | 6.86 | 41.91 | -0.638 |
| BhbZIP50 | Bhi10G001078 | chr10 | 32950605:32953718 | 278 | 7.17 | 31.85 | -0.947 |
| BhbZIP51 | Bhi10G001457 | chr10 | 46004337:46007733 | 273 | 6.26 | 29.84 | -0.729 |
| BhbZIP52 | Bhi10G001591 | chr10 | 49402470:49404070 | 160 | 5.37 | 17.61 | -0.475 |
| BhbZIP53 | Bhi11G001061 | chr11 | 37143787:37147045 | 142 | 9.42 | 15.89 | -0.508 |
| BhbZIP54 | Bhi11G001648 | chr11 | 55713254:55717815 | 519 | 6.85 | 58.01 | -0.741 |
| BhbZIP55 | Bhi11G001911 | chr11 | 62710703:62714289 | 442 | 8.64 | 47.37 | -0.587 |
| BhbZIP56 | Bhi11G002213 | chr11 | 71682473:71691648 | 514 | 6.73 | 57.86 | -0.654 |
| BhbZIP57 | Bhi11G002318 | chr11 | 74922564:74931505 | 468 | 8.41 | 51.65 | -0.55 |
| BhbZIP58 | Bhi11G002347 | chr11 | 75954267:75960158 | 763 | 6.39 | 82.68 | -0.565 |
| BhbZIP59 | Bhi12G000925 | chr12 | 31933500:31935616 | 147 | 5.75 | 17.22 | -0.839 |
| BhbZIP60 | BhiUN613G6 | Contig613 | 139383:145977 | 387 | 8.77 | 42.51 | -0.791 |
| BhbZIP61 | BhiUN916G5 | Contig916 | 105521:109140 | 421 | 6.86 | 47.52 | -0.747 |

**Table S2** Secondary structure and subcellular localization of the 61 *BhbZIP* gene family members.

| Gene name | Alpha helix | Extended trand | Beta turn | Random coil | Subcellular localization |
| --- | --- | --- | --- | --- | --- |
| BhbZIP1 | 26.14% | 4.32% | 2.40% | 67.15% | Nucleus. |
| BhbZIP2 | 33.41% | 4.06% | 1.43% | 61.10% | Nucleus. |
| BhbZIP3 | 36.56% | 4.84% | 1.94% | 56.66% | Nucleus. |
| BhbZIP4 | 35.98% | 2.91% | 0.53% | 60.58% | Nucleus. |
| BhbZIP5 | 51.53% | 3.07% | 1.84% | 43.56% | Nucleus. |
| BhbZIP6 | 60.26% | 2.65% | 3.97% | 33.11% | Nucleus. |
| BhbZIP7 | 57.59% | 7.43% | 2.79% | 32.20% | Nucleus. |
| BhbZIP8 | 36.63% | 4.40% | 2.20% | 56.78% | Nucleus. |
| BhbZIP9 | 72.86% | 2.86% | 5.00% | 19.29% | Nucleus. |
| BhbZIP10 | 40.48% | 5.71% | 2.86% | 50.95% | Nucleus. |
| BhbZIP11 | 25.27% | 4.67% | 1.65% | 68.41% | Nucleus. |
| BhbZIP12 | 66.37% | 6.61% | 1.50% | 25.53% | Nucleus. |
| BhbZIP13 | 71.32% | 2.21% | 1.47% | 25.00% | Nucleus. |
| BhbZIP14 | 31.87% | 9.73% | 1.70% | 56.69% | Nucleus. |
| BhbZIP15 | 67.61% | 2.84% | 1.14% | 28.41% | Nucleus. |
| BhbZIP16 | 52.53% | 0.63% | 0.63% | 46.20% | Nucleus. |
| BhbZIP17 | 37.61% | 6.73% | 2.75% | 52.91% | Nucleus. |
| BhbZIP18 | 33.02% | 5.92% | 1.56% | 59.50% | Nucleus. |
| BhbZIP19 | 41.30% | 5.59% | 1.55% | 51.55% | Nucleus. |
| BhbZIP20 | 37.71% | 6.29% | 2.57% | 53.43% | Nucleus. |
| BhbZIP21 | 50.75% | 10.71% | 2.57% | 35.97% | Nucleus. |
| BhbZIP22 | 66.45% | 6.58% | 1.32% | 25.66% | Nucleus. |
| BhbZIP23 | 59.00% | 8.00% | 2.75% | 30.25% | Nucleus. |
| BhbZIP24 | 20.20% | 5.24% | 0.50% | 74.06% | Nucleus. |
| BhbZIP25 | 47.58% | 2.60% | 1.12% | 48.70% | Nucleus. |
| BhbZIP26 | 31.30% | 9.29% | 1.22% | 58.19% | Nucleus. |
| BhbZIP27 | 27.94% | 11.40% | 1.47% | 59.19% | Nucleus. |
| BhbZIP28 | 28.98% | 4.95% | 1.06% | 65.02% | Nucleus. |
| BhbZIP29 | 30.21% | 3.82% | 1.56% | 64.41% | Nucleus. |
| BhbZIP30 | 53.63% | 3.91% | 0.00% | 42.46% | Nucleus. |
| BhbZIP31 | 37.79% | 2.53% | 0.92% | 58.76% | Nucleus. |
| BhbZIP32 | 34.99% | 4.96% | 1.17% | 58.89% | Nucleus. |
| BhbZIP33 | 43.01% | 2.80% | 1.05% | 53.15% | Nucleus. |
| BhbZIP34 | 24.35% | 5.44% | 0.47% | 69.74% | Nucleus. |
| BhbZIP35 | 68.88% | 3.93% | 1.51% | 25.68% | Nucleus. |
| BhbZIP36 | 56.50% | 3.00% | 0.00% | 40.50% | Nucleus. |
| BhbZIP37 | 57.47% | 3.80% | 2.28% | 36.46% | Nucleus. |
| BhbZIP38 | 48.82% | 9.48% | 3.32% | 38.39% | Nucleus. |
| BhbZIP39 | 67.86% | 2.14% | 2.86% | 27.14% | Nucleus. |
| BhbZIP40 | 33.11% | 2.72% | 0.68% | 63.49% | Nucleus. |
| BhbZIP41 | 35.14% | 7.14% | 2.29% | 55.43% | Nucleus. |
| BhbZIP42 | 63.28% | 9.04% | 2.82% | 24.86% | Nucleus. |
| BhbZIP43 | 41.87% | 11.82% | 0.49% | 45.81% | Nucleus. |
| BhbZIP44 | 46.22% | 8.40% | 3.08% | 42.30% | Nucleus. |
| BhbZIP45 | 64.71% | 7.84% | 0.00% | 27.45% | Nucleus. |
| BhbZIP46 | 36.11% | 2.16% | 2.47% | 59.26% | Nucleus. |
| BhbZIP47 | 44.35% | 2.90% | 0.87% | 51.88% | Nucleus. |
| BhbZIP48 | 46.44% | 7.32% | 3.08% | 43.16% | Nucleus. |
| BhbZIP49 | 30.43% | 4.35% | 1.53% | 63.68% | Nucleus. |
| BhbZIP50 | 37.41% | 4.68% | 2.52% | 55.40% | Nucleus. |
| BhbZIP51 | 28.94% | 9.16% | 4.03% | 57.88% | Nucleus. |
| BhbZIP52 | 65.00% | 1.88% | 1.25% | 31.87% | Nucleus. |
| BhbZIP53 | 66.20% | 3.52% | 1.41% | 28.87% | Nucleus. |
| BhbZIP54 | 33.33% | 4.24% | 0.77% | 61.66% | Nucleus. |
| BhbZIP55 | 32.35% | 4.75% | 1.81% | 61.09% | Nucleus. |
| BhbZIP56 | 49.42% | 6.61% | 1.95% | 42.02% | Nucleus. |
| BhbZIP57 | 52.78% | 7.05% | 1.50% | 38.68% | Nucleus. |
| BhbZIP58 | 17.56% | 11.80% | 1.83% | 68.81% | Nucleus. |
| BhbZIP59 | 62.59% | 3.40% | 1.36% | 32.65% | Nucleus. |
| BhbZIP60 | 43.41% | 1.81% | 1.29% | 53.49% | Nucleus. |
| BhbZIP61 | 33.49% | 6.41% | 1.43% | 58.67% | Nucleus. |

**Table S3** Protein–Protein Interaction of online STRING

| Gene | Group | protein families | multiple sequences |
| --- | --- | --- | --- |
| Bhi01M001706 | S | NOG243340 | bZIP3 |
| Bhi02M001384 | A | NOG243340 | AREB3 |
| Bhi04M000330 | A | NOG243340 | ABF2 |
| Bhi04M001290 | A | NOG243340 | AREB3 |
| Bhi04M001359 | A | NOG243340 | AT5G44080 |
| Bhi04M001617 | S | NOG243340 | bZIP3 |
| Bhi05M000555 | A | NOG243340 | GBF4 |
| Bhi05M000928 | A | NOG243340 | ABF3 |
| Bhi06M000246 | S | NOG243340 | bZIP3 |
| Bhi07M001174 | S | NOG243340 | bZIP42 |
| Bhi09M002211 | A | NOG243340 | AREB3 |
| Bhi12M000925 | S | NOG243340 | bZIP3 |
| Bhi04M000003 | D | NOG06126 | AHBP-1B |
| Bhi07M001014 | D | NOG06127 | AHBP-1B |
| Bhi11M002318 | D | NOG06128 | AHBP-1B |
| Bhi01M000318 | G | NOG06792 | GBF3 |
| Bhi03M000984 | G | NOG06792 | bZIP16 |
| Bhi05M000290 | G | NOG06792 | bZIP68 |
| Bhi07M000824 | G | NOG06792 | bZIP16 |
| Bhi01M001186 | E | NOG01780 | AT1G58110 |
| Bhi07M000619 | E | NOG01780 | AT5G04840 |
| Bhi09M002846 | E | NOG01780 | BZIP34 |
| Bhi10M001078 | E | NOG01780 | BZIP34 |
| BhiUN916M5 | E | NOG01780 | AT1G58110 |
| Bhi05M001531 | I | NOG258298 | AT4G38900 |
| Bhi05M001533 | I | NOG258298 | AT4G38900 |
| Bhi01M000868 | I | NOG05447 | AT1G06070 |
| Bhi01M000872 | I | NOG05447 | AT1G06070 |
| Bhi01M001913 | D | NOG03329 | TGA1 |
| Bhi04M002048 | D | NOG03329 | TGA7 |
| Bhi04M001577 | D | NOG259341 | PAN |
| Bhi07M000420 | I | NOG258070 | AT2G40620 |
| Bhi10M000691 | I | NOG258070 | AT2G40620 |
| Bhi08M000051 | D | NOG02544, KOG0167 | TGA9 |
| Bhi10M000031 | D | NOG02544, KOG0167 | TGA9 |
| Bhi06M001517 | C | NOG03282 | BZO2H3 |
| BhiUN613M6 | C | NOG03282 | BZO2H3 |
| Bhi05M001473 | F | NOG08178 | bZIP23 |
| Bhi10M001457 | F | NOG08178 | bZIP23 |
| Bhi11M002213 | D | NOG258315 | TGA10 |
| Bhi04M001371 | I | NOG258335 | AT1G06070 |
| Bhi09M001219 | I | NOG258335 | AT2G40620 |
| Bhi01M001873 | S | NOG270019 | BZIP53 |
| Bhi09M000681 | S | NOG270019 | BZIP53 |
| Bhi09M001870 | S | NOG270019, NOG269807 | bZIP44 |
| Bhi11M001061 | S | NOG270019 | BZIP53 |
| Bhi03M000687 | S | NOG269807 | bZIP44 |
| Bhi04M000176 | S | NOG269807 | bZIP2 |
| Bhi10M001591 | S | NOG269807 | bZIP44 |
| Bhi11M001911 | A | NOG257560 | ABI5 |
| Bhi11M002347 | B | NOG39651, NOG01724 | BZIP17 |
| Bhi10M000025 | A | NOG259163 | DPBF2 |
| Bhi04M000896 | C | NOG07729 | BZIP9 |
| Bhi04M000795 | H | KOG1414 | HY5 |
| Bhi09M000917 | C | NOG10040 | BZO2H1 |
| Bhi03M000814 | S | NOG262435 | bZIP6 |
| Bhi04M000415 | S | NOG262435, NOG22462 | bZIP6 |
| Bhi09M002310 | K | COG2801 | BZIP60 |
| Bhi11M001648 | J | KOG0724, NOG08719 | AT1G19490 |
| Bhi09M002631 | S | NOG270233 | bZIP6 |
| Bhi08M000880 | S | NA | bZIP3 |

**Table S4** Gene duplication type of the *BhbZIP* gene family

| gene | duplication type | gene | duplication type |
| --- | --- | --- | --- |
| Bhi01M000318 | Dispersed | Bhi01M000868 | Proximal |
| Bhi01M001186 | Dispersed | Bhi01M000872 | Proximal |
| Bhi01M001873 | Dispersed | Bhi05M001531 | Proximal |
| Bhi01M001913 | Dispersed | Bhi05M001533 | Proximal |
| Bhi03M000984 | Dispersed | Bhi02M001384 | Transposed |
| Bhi04M000330 | Dispersed | Bhi04M000003 | Transposed |
| Bhi04M000896 | Dispersed | Bhi04M001290 | Transposed |
| Bhi04M001371 | Dispersed | Bhi04M001359 | Transposed |
| Bhi04M002048 | Dispersed | Bhi04M001577 | Transposed |
| Bhi05M000290 | Dispersed | Bhi04M001617 | Transposed |
| Bhi05M000928 | Dispersed | Bhi05M000555 | Transposed |
| Bhi06M000246 | Dispersed | Bhi07M000420 | Transposed |
| Bhi06M001517 | Dispersed | Bhi09M002211 | Transposed |
| Bhi07M000619 | Dispersed | Bhi11M001648 | Transposed |
| Bhi07M000824 | Dispersed | Bhi12M000925 | Transposed |
| Bhi07M001014 | Dispersed | Bhi01M001706 | WGD |
| Bhi07M001174 | Dispersed | Bhi03M000687 | WGD |
| Bhi08M000051 | Dispersed | Bhi03M000814 | WGD |
| Bhi09M000917 | Dispersed | Bhi04M000176 | WGD |
| Bhi09M001219 | Dispersed | Bhi04M000415 | WGD |
| Bhi09M002310 | Dispersed | Bhi04M000795 | WGD |
| Bhi09M002846 | Dispersed | Bhi05M001473 | WGD |
| Bhi10M000025 | Dispersed | Bhi08M000880 | WGD |
| Bhi10M000031 | Dispersed | Bhi09M000681 | WGD |
| Bhi10M000691 | Dispersed | Bhi09M001870 | WGD |
| Bhi10M001078 | Dispersed | Bhi09M002631 | WGD |
| Bhi11M001911 | Dispersed | Bhi10M001457 | WGD |
| Bhi11M002213 | Dispersed | Bhi10M001591 | WGD |
| Bhi11M002318 | Dispersed | Bhi11M001061 | WGD |
| Bhi11M002347 | Dispersed | |  |

**Table S5** Selective pressure analysis of the *BhbZIP* gene family.

| Sequence | Ka | Ks | Ka/Ks | P-Value (Fisher) | subgroup |
| --- | --- | --- | --- | --- | --- |
| Bhi10M001457-Bhi05M001473 | 0.162562 | 1.62428 | 0.100083 | 1.40E-83 | F |
| Bhi03M000687-Bhi04M000176 | 0.318781 | 3.33456 | 0.095599 | 0 | S |
| Bhi03M000687-Bhi09M001870 | 0.366946 | 3.49006 | 0.10514 | 0 | S |
| Bhi04M000176-Bhi09M001870 | 0.334118 | 3.08967 | 0.108141 | 0 | S |
| Bhi04M000415-Bhi09M002631 | 0.349425 | 1.84631 | 0.189255 | 3.34E-46 | S |
| Bhi03M000814-Bhi04M000415 | 0.486807 | 2.67982 | 0.181656 | 0 | S |
| Bhi03M000814-Bhi09M002631 | 0.789943 | 1.66863 | 0.473409 | 4.57E-20 | S |
| Bhi01M001706-Bhi08M000880 | 0.563839 | 2.65952 | 0.212008 | 0 | S |
| Bhi10M001591-Bhi04M000176 | 0.316717 | 3.57502 | 0.088592 | 0 | S |
| Bhi11M001061-Bhi09M000681 | 0.34871 | 2.07614 | 0.167961 | 0 | S |
| Bhi01M000868-Bhi01M000872 | 0.125671 | 0.540027 | 0.232711 | 1.88E-31 | I |
| Bhi05M001531-Bhi05M001533 | 0.065622 | 0.482551 | 0.135989 | 7.01E-53 | I |

**Table S6** Collinear *bZIP* gene pairs between species

| gene-pair | |
| --- | --- |
| **Csa-Bhi** | |
| CsaV3_1G041950-Bhi02M001384 | CsaV3_1G014770-Bhi08M000051 |
| CsaV3_2G025950-Bhi03M000814 | CsaV3_1G006310-Bhi08M000880 |
| CsaV3_2G029010-Bhi04M000176 | CsaV3_4G009880-Bhi08M000880 |
| CsaV3_2G026630-Bhi04M000330 | CsaV3_5G033640-Bhi08M000880 |
| CsaV3_2G025950-Bhi04M000415 | CsaV3_7G029950-Bhi09M000681 |
| CsaV3_2G031180-Bhi04M000896 | CsaV3_2G004160-Bhi09M000917 |
| CsaV3_1G019640-Bhi04M001371 | CsaV3_7G027650-Bhi09M000917 |
| CsaV3_2G033550-Bhi04M002048 | CsaV3_7G025120-Bhi09M001219 |
| CsaV3_3G048810-Bhi05M000290 | CsaV3_2G029010-Bhi09M001870 |
| CsaV3_3G001310-Bhi05M000290 | CsaV3_3G045480-Bhi09M001870 |
| CsaV3_2G016100-Bhi05M001473 | CsaV3_6G046670-Bhi09M001870 |
| CsaV3_2G004160-Bhi06M001517 | CsaV3_4G028620-Bhi09M002211 |
| CsaV3_5G010910-Bhi07M000420 | CsaV3_4G037700-Bhi09M002310 |
| CsaV3_5G035190-Bhi07M000619 | CsaV3_2G025950-Bhi09M002631 |
| CsaV3_3G001310-Bhi07M000824 | CsaV3_4G035230-Bhi09M002631 |
| CsaV3_5G036870-Bhi07M001014 | CsaV3_6G044300-Bhi09M002631 |
| CsaV3_1G006310-Bhi07M001174 | CsaV3_4G033330-Bhi09M002846 |
| CsaV3_5G033640-Bhi07M001174 |  |
| **Ath-Bhi** | |
| AT2G46270-Bhi01M000318 | AT5G38800-Bhi06M000246 |
| AT4G01120-Bhi01M000318 | AT5G15830-Bhi06M000246 |
| AT3G30530-Bhi01M001706 | AT2G40620-Bhi07M000420 |
| AT5G38800-Bhi01M001706 | AT5G04840-Bhi07M000619 |
| AT5G10030-Bhi01M001913 | AT2G35530-Bhi07M000824 |
| AT5G65210-Bhi01M001913 | AT1G13600-Bhi07M001174 |
| AT2G18160-Bhi03M000687 | AT2G04038-Bhi07M001174 |
| AT4G34590-Bhi03M000687 | AT1G08320-Bhi08M000051 |
| AT2G22850-Bhi03M000814 | AT3G30530-Bhi08M000880 |
| AT3G49760-Bhi03M000814 | AT5G60830-Bhi08M000880 |
| AT4G37730-Bhi03M000814 | AT5G15830-Bhi08M000880 |
| AT4G36730-Bhi03M000984 | AT3G62420-Bhi09M000681 |
| AT1G75390-Bhi04M000176 | AT4G02640-Bhi09M000917 |
| AT1G45249-Bhi04M000330 | AT1G75390-Bhi09M001870 |
| AT3G19290-Bhi04M000330 | AT3G49760-Bhi09M002631 |
| AT3G49760-Bhi04M000415 | AT2G36270-Bhi10M000025 |
| AT5G11260-Bhi04M000795 | AT3G44460-Bhi10M000025 |
| AT1G32150-Bhi05M000290 | AT2G16770-Bhi10M001457 |
| AT2G35530-Bhi05M000290 | AT4G35040-Bhi10M001457 |
| AT3G19290-Bhi05M000928 | AT1G75390-Bhi10M001591 |
| AT2G16770-Bhi05M001473 | AT2G18160-Bhi10M001591 |
| AT4G35040-Bhi05M001473 | AT4G34590-Bhi10M001591 |
| AT2G21230-Bhi05M001531 | AT3G62420-Bhi11M001061 |
| AT4G38900-Bhi05M001531 | AT2G36270-Bhi11M001911 |
| AT3G30530-Bhi06M000246 |  |
| **Vit-Bhi** | |
| VIT_02s0025g01020-Bhi01M000318 | VIT_13s0073g00430-Bhi07M000619 |
| VIT_15s0046g01440-Bhi01M000318 | VIT_02s0012g02250-Bhi07M000824 |
| VIT_12s0035g00620-Bhi01M001186 | VIT_13s0084g00660-Bhi07M001014 |
| VIT_14s0083g00700-Bhi01M001706 | VIT_01s0010g00930-Bhi07M001174 |
| VIT_04s0023g02430-Bhi01M001873 | VIT_06s0080g00360-Bhi08M000051 |
| VIT_18s0001g13040-Bhi01M001873 | VIT_14s0083g00700-Bhi08M000880 |
| VIT_07s0031g01320-Bhi01M001913 | VIT_07s0005g01450-Bhi09M000681 |
| VIT_18s0001g04470-Bhi01M001913 | VIT_14s0060g01210-Bhi09M000681 |
| VIT_04s0023g02430-Bhi03M000687 | VIT_07s0141g00170-Bhi09M000917 |
| VIT_18s0001g13040-Bhi03M000687 | VIT_04s0023g02430-Bhi09M001870 |
| VIT_18s0001g08710-Bhi03M000814 | VIT_18s0001g13040-Bhi09M001870 |
| VIT_04s0023g01360-Bhi03M000984 | VIT_18s0122g00500-Bhi09M002310 |
| VIT_18s0001g12120-Bhi03M000984 | VIT_18s0001g08710-Bhi09M002631 |
| VIT_04s0023g02430-Bhi04M000176 | VIT_12s0028g02590-Bhi09M002846 |
| VIT_18s0001g13040-Bhi04M000176 | VIT_19s0014g01780-Bhi09M002846 |
| VIT_03s0063g00310-Bhi04M000330 | VIT_06s0080g00340-Bhi10M000025 |
| VIT_18s0001g10450-Bhi04M000330 | VIT_08s0007g03420-Bhi10M000025 |
| VIT_18s0001g08710-Bhi04M000415 | VIT_06s0080g00360-Bhi10M000031 |
| VIT_04s0008g05210-Bhi04M000795 | VIT_06s0004g08070-Bhi10M000691 |
| VIT_04s0008g02750-Bhi04M000896 | VIT_12s0028g02590-Bhi10M001078 |
| VIT_18s0001g04470-Bhi04M002048 | VIT_03s0038g02420-Bhi10M001457 |
| VIT_02s0012g02250-Bhi05M000290 | VIT_03s0038g04450-Bhi10M001591 |
| VIT_03s0063g00310-Bhi05M000928 | VIT_04s0023g02430-Bhi10M001591 |
| VIT_03s0038g02420-Bhi05M001473 | VIT_18s0001g13040-Bhi10M001591 |
| VIT_03s0038g00860-Bhi05M001531 | VIT_07s0005g01450-Bhi11M001061 |
| VIT_18s0001g13740-Bhi05M001531 | VIT_14s0060g01210-Bhi11M001061 |
| VIT_01s0010g00930-Bhi06M000246 | VIT_08s0007g03420-Bhi11M001911 |
| VIT_14s0083g00700-Bhi06M000246 | VIT_08s0007g06160-Bhi11M002213 |
| VIT_07s0141g00170-Bhi06M001517 | VIT_08s0007g05170-Bhi11M002318 |
| VIT_14s0030g02200-Bhi06M001517 | VIT_08s0040g00870-Bhi11M002347 |
| **Os-Bhi** | |
| Os01t0658900-Bhi01M000318 | Os12t0152900-Bhi08M000051 |
| Os08t0472000-Bhi04M000330 | Os05t0443900-Bhi08M000051 |
| Os09t0456200-Bhi04M000330 | Os05t0129300-Bhi09M000681 |
| Os01t0174000-Bhi04M000795 | Os03t0796900-Bhi09M000917 |
| Os03t0336200-Bhi07M000420 | Os11t0152700-Bhi10M000031 |
| Os01t0859500-Bhi08M000051 |  |

**Table S7** Primers used in this study.

| Primer name |  | Primer sequence |
| --- | --- | --- |
| BhbZIP58-qPCR | F | CCCAGAGAAGCTGGTGACAG |
|  | R | CTGAGCGGGGAAGAACACAT |
| Actin (Bhi10G001911) | F | ATGTTCACAACCACTGCCGA |
|  | R | GTCGAGCGCAACATAAGCAA |
| AtActin | F | TAACAGGGAGAAGATGACTCAGATCA |
|  | R | AAGATCAAGACGAAGGATAGCATGAG |
| BhbZIP58 gene clone | F1 | ATGGCCGACCCATTTGAGGTCG |
|  | R1 | GAAGGATGAGGATACATCCCAGGAGGAGG |
|  | F2 | AGGTCTAAGACAGCAGCTGAGTAGTAGT |
|  | R2 | TTAAGTAGACACGAGATGAGGACCTGAG |
| pSuper1300-BhiZIP58 | F | caaatcgactctagaaagcttATGGCCGACCCATTTGAGG |
|  | R | cctcgcccttgctcaccatggtaccAGTAGACACGAGATGAGGACCT |
| pYES2-BhbZIP58 | F | gccgccagtgtgctggaattcATGGCCGACCCATTTGAGG |
|  | R | ccctctagatgcatgctcgagTTAAGTAGACACGAGATGAGGACCTG |
| pBI121-BhbZIP58 | F | acgggggactctagaggatccATGGCCGACCCATTTGAGG |
|  | R | cgatcggggaaattcgagctcTTAAGTAGACACGAGATGAGGACCTG |
| pGBKT7-BD-BhbZIP58 | F | tcagaggaggacctgcatatgATGGCCGACCCATTTGAGG |
|  | R | tttcggcctccatggccatatgTTAAGTAGACACGAGATGAGGACCTG |
